# The pathogenic human Torsin A in *Drosophila* activates the unfolded protein response and increases susceptibility to oxidative stress

**DOI:** 10.1186/s12864-015-1518-0

**Published:** 2015-04-23

**Authors:** A-Young Kim, Jong Bok Seo, Won-tae Kim, Hee Jeong Choi, Soo-Young Kim, Genevieve Morrow, Robert M Tanguay, Hermann Steller, Young Ho Koh

**Affiliations:** ILSONG Institute of Life Science, Hallym University, 1605-4 Gwanyangdong, Dongan-gu, Anyang, Gyeonggido 431-060 Republic of Korea; Department of Biomedical Gerontology, Graduate School of Hallym University, Chuncheon, Gangwon-do 200-702 Republic of Korea; Korea Basic Science Institute, Sungbuk-gu, Seoul 136-713 Republic of Korea; National Academy of Agricultural Science, Rural Development Administration, Suwon, 441-707 Republic of Korea; Department of Molecular Biology, Medical Biochemistry & Pathology, Université Laval, Québec, Qc G1V 0A6 Canada; Howard Hughes Medical Institute, the Rockefeller University, New York, NY 10065 USA

**Keywords:** DYT1 dystonia, Bip, HSC3, HSP22, Xbp1

## Abstract

**Background:**

Dystonia1 (DYT1) dystonia is caused by a glutamic acid deletion (ΔE) mutation in the gene encoding Torsin A in humans (HTorA). To investigate the unknown molecular and cellular mechanisms underlying DYT1 dystonia, we performed an unbiased proteomic analysis.

**Results:**

We found that the amount of proteins and transcripts of an Endoplasmic reticulum (ER) resident chaperone Heat shock protein cognate 3 (HSC3) and a mitochondria chaperone Heat Shock Protein 22 (HSP22) were significantly increased in the HTorA^ΔE^– expressing brains compared to the normal HTorA (HTorA^WT^) expressing brains. The physiological consequences included an increased susceptibility to oxidative and ER stress compared to normal HTorA^WT^ flies. The alteration of transcripts of Inositol-requiring enzyme-1 (IRE1)-dependent spliced X box binding protein 1(Xbp1), several ER chaperones, a nucleotide exchange factor, Autophagy related protein 8b (ATG8b) and components of the ER associated degradation (ERAD) pathway and increased expression of the Xbp1-enhanced Green Fluorescence Protein (eGFP) in HTorA^ΔE^ brains strongly indicated the activation of the unfolded protein response (UPR). In addition, perturbed expression of the UPR sensors and inducers in the HTorA^ΔE^*Drosophila* brains resulted in a significantly reduced life span of the flies. Furthermore, the types and quantities of proteins present in the anti-HSC3 positive microsomes in the HTorA^ΔE^ brains were different from those of the HTorA^WT^ brains.

**Conclusion:**

Taken together, these data show that HTorA^ΔE^ in *Drosophila* brains may activate the UPR and increase the expression of HSP22 to compensate for the toxic effects caused by HTorA^ΔE^ in the brains.

**Electronic supplementary material:**

The online version of this article (doi:10.1186/s12864-015-1518-0) contains supplementary material, which is available to authorized users.

## Background

The appearance of misfolded and aggregated proteins in specific regions of the brain is one of the common pathological hallmarks of various progressive neurological disorders in humans, such as early onset torsion dystonia (DYT1 dystonia) [1], Alzheimer’s disease, Amyotrophic lateral sclerosis, Parkinson’s disease, various polyglutamine diseases and prion disease [2]. A variety of evidence suggests that accumulated misfolded proteins are key players in the onset and progression of these neurological disorders [1,2]. The endoplasmic reticulum (ER) is a sub-cellular organelle where secretory and membrane proteins are synthesised, modified, correctly folded and assembled prior to transit to the cell surface or to intracellular organelles. Perturbations of ER function, caused by various stressors, may result in the underlying deposition of intra- and/or extracellularly accumulated misfolded proteins in the brain, which has been associated with various neurological disorders [3,4]. The known ER stressors include physiological stress, pathological stress and environmental stress [5].

To sense and respond to ER stressors, eukaryotic cells have developed a group of evolutionarily conserved signal transduction pathways known as the unfolded protein response (UPR), which is characterised by transcriptional up-regulation of ER resident chaperones, selective inhibition of translation, and activation of ER-associated degradation (ERAD) [6]. Under physiological conditions, the ER resident chaperone BiP, also known as Glucose-regulated protein 78 (GRP78), and Heat shock 70 kDa protein 5 (HSPA5) are bound to three UPR inducers, such as the pancreatic ER kinase (PERK), the inositol-requiring enzyme 1 (IRE1) and the activating transcription factor 6 (ATF-6). When unfolded proteins accumulate in the ER lumen, BiP binds to unfolded stretches in the proteins and becomes dissociated from the three UPR inducers, which subsequently activate the UPR that induces expression of BiP and other ER chaperones. If the increased BiP expression is sufficient for restoring ER function, it inactivates the UPR by binding to the three UPR inducers [4,6,7]. However, prolonged activation of the UPR by mild or strong ER stresses may result in the adaptation and survival of cells under these conditions or cell death [8]. Recent studies have shown that the prolonged activation of the UPR may contribute to the pathogenesis of autosomal dominant retinitis pigmentosa (ADRP) [9], Alzheimer’s disease [10], Parkinson’s disease [5,11], and Amyotrophic lateral sclerosis [12].

DYT1 dystonia is the most common and severe form of dystonia caused by mutations in the DYT1 gene encoding Torsin A in humans (HTorA). DYT1 patients show severe twisting movements and abnormal postures caused by involuntary muscle contractions. The loss of one of a pair of glutamic acid residues in HTorA (HTorA^ΔΕ^) has been identified in most patients with this disorder [13]. Only 30-40% of heterozygous individuals manifest severe symptoms, suggesting that the ΔE mutation in HTorA may increase an individual’s susceptibilities to other genetic and/or environmental risk factors required for the development of this disorder [14,15]. Amino acid sequence comparisons revealed that HTorA is a member of a family of ATPases associated with a variety of cellular activities (AAA+ ATPase) and as such, may play a pivotal role in the assembly, disassembly, degradation and trafficking of other proteins [13,16]. Moreover, recent studies have shown that the secretory pathway in fibroblasts acquired from DYT1 dystonia patients is impaired [17], and the impaired secretory pathway in those cells is rescued by the down-regulation of HTorA^ΔE^ via the expression of an allele specific small interfering (si) RNA of HTorA^ΔE^ [18].

Another consequence of the ΔE mutation on HTorA at the subcellular level is the redistribution of HTorA^ΔE^ to the nuclear envelope (NE) that results in the appearance of perinuclear membranous inclusions in DYT1 dystonia fibroblasts [19], TorsinA^ΔE^ knock-in mouse brains [20], and transgenic mice over-expressing HTorA^ΔE^ in the brain [21]. At the molecular level, interactions between the HTorA^WT^ and its interaction partners, including the Lamina-associated polypeptide 1 (LAP1) in the NE, the luminal domain like LAP1 (LULL1) in the ER [22,23], the NE network-associated protein Vimentin [24], and Kinesin light chain 1 (KLC1) [25,26] are abolished in the HTorA^ΔE^. Recently, it has been shown that ultrastructural defects in the neuronal NE in the LAP1 knock out mouse are similar to those observed in the Tor1A^ΔE^ knock-in mouse, suggesting that HTorA and LAP1 may be present in the same signal transduction pathway that regulates normal development of the neuronal NE [26]. These reports suggest that DYT1 pathogenesis could be the consequence of altered transcriptional regulation caused by abnormal interactions of HTorA^ΔE^ with nuclear envelope proteins and/or a loss of function of HTorA^WT^ in the ER that would impair certain neuron-specific signalling pathways.

To investigate how transcriptional or translational alterations contribute to the pathogenesis of DYT1, unbiased genomics and proteomic analyses were recently performed using *in vitro* and *in vivo* DYT1 models. The expression of HTorA^ΔE^ in neuronal cell lines did not induce transcriptional alterations; however, expression changes of proteins involved with energy metabolism and the redox state were detected in this model [27]. A recent study revealed that the expression of several genes involved in the development and function of the nervous system, cytoskeleton organisation and biogenesis, cell adhesion, G-protein-receptor signalling and the vesicle mediated trafficking pathway were altered when transcriptional profiles in peripheral blood cells from DYT1 dystonia patients were compared with those of HTorA^ΔE^ carriers [28].

*Drosophila* has been extensively utilised as a model to investigate the molecular and cellular aetiologies underlying diverse neurological diseases in humans. We have shown that HTorA^ΔE^ but not HTorA^WT^ expressed in *Drosophila* induced protein aggregates near the NE, caused defects at synaptic terminals, and increased the flies’ susceptibility to environmental stress [29,30]. In this study, we gained further insights into the molecular and cellular consequences of HTorA^ΔE^ in *Drosophila* brains by performing an unbiased 2-dimensional electrophoresis analysis that demonstrated that Heat shock protein cognate 3 and Heat shock protein 22 were dysregulated with the expression of HTorA^ΔE^. In addition, we performed biochemical, behavioural, cellular and molecular biological, genetic, pharmacological and proteomics profiling analyses to provide several lines of evidence supporting the observation that UPR activation and increased susceptibility to oxidative stress were the consequences of HTorA^ΔE^ expression in *Drosophila*.

## Methods

### Fly genetics

UAS-HTorA^WT^, UAS-HTorA^ΔE^, Tubulin-Gal4, C155-Gal4 [29,30], *xbp1*^*K13803*^/CyO and UAS-Xbp1-eGFP [31] were previously characterised. Tubulin-Gal4, UAS-HTorA^WT^/TM6b Tb^1^ and UAS-HTorA^ΔE^/CyO, P{GAL4-Kr.C}DC3, P{UAS-GFP.S65T}DC7 flies were generated via chromosome recombination. RNAi flies for Xbp1, HSC3, Activation transcription factor 6 (ATF6) and Pancreatic elF-2a kinase (PEK) and *X box binding protein −1(xbp1*)^K13803^/CyO were obtained from the Vienna *Drosophila* RNAi Center (VDRC) and the Bloomington stock centre. UAS-Actin-GFP (Act-eGFP) flies were obtained from Bloomington stock centre. UAS-DTor-cDNA (DTor) flies were generated by cloning full length DTor-cDNA into pUAST germ line transformation vectors. Flies were reared on a standard *Drosophila* medium in a 16 h light – 8 h dark cycle at 25 ± 1°C and 60 ± 1% relative humidity.

### 2-Dimensional electrophoresis

As described in Koh et al. [29], Tubulin-Gal4/UAS-HTorA^WT^ and UAS-HTorA^ΔE^/+; Tubulin-Gal4/+ flies collected from three different independent crosses were raised at 30°C for accelerating aging. Proteins were extracted from 10 day old adult fly heads by grinding for 5 min using a manual pestle in 100 μL lysis buffer (8.0 M urea, 18 mM DTT, 4% (w/v) CHAPS, 40 mM Tris–HCl (pH 8.0), 10 mM EDTA, 0.5% IPG buffer (pH4-7, GE healthcare, Germany) with protease inhibitor cocktail (Roche Diagnostics, GmbH, Germany). Next, 300 μL of lysis buffer was added, the solution was centrifuged at 15,000 rpm (Vision scientific Co., Korea) for 10 min and the supernatants were collected. The concentrations of proteins were quantified using the Bradford assay. A total of 200 μg of proteins were applied on immobilised linear gradient strips (pH 4–7) using the IPGphor system (Amersham Pharmacia Biotech, Uppsala, Sweden). After rehydration for 12 hr, focusing was performed in the following three steps: 200 V for 1 hr, 500 V for 1 hr and a final phase of 8,000 V for 8 hr. After the reduction and alkylation, proteins were achieved by incubating strips for 15 min while shaking in 1.5 M Tris–HCl buffer (pH 8.8, 10% SDS, 87% glycerol, 6 M urea, 64.8 mM DTT), and the second dimension was run on a 12% poly-acrylamide SDS gel using an Ethan Dalt electrophoresis kit (Amersham Pharmacia Biotech., Uppsala, Sweden). Two-D gels stained with 0.1% Coomassie Brilliant Blue R250 were scanned with a PowerLook III image scanner (UMAX data system, Hsinchu, Taiwan) and analysed using Progenesis Editor software (Nonlinear Dynamics Ltd., Newcastle, UK) with the exclusion filter set manually. The total number of protein spots on the 6 gels ranged from 1415 to 2534 (WT1, 1415; WT2, 1927; WT3, 2534; ΔE1, 1444, ΔE2, 1928, and ΔE3, 2178). The normalised volumes of the protein spots from three different comparisons were analysed using one-way Anova (threshold of significance; P < 0.05) to select protein spots for subsequent analyses.

### Identification of proteins in differentially expressed protein spots

Seven differentially expressed spots were excised from the gels, washed with 10 mM NH_4_HCO_3_ and 50% CH_3_CN (Sigma), and digested in a buffer containing 50 mM NH_4_HCO_3_, 5 mM CaCl_2_, and 12.5 ng/ml Trypsin Gold (Promega, Madison, WI, USA) at 37°C for 12–16 h. The digested peptides were recovered by extraction twice with 50 mM NH_4_HCO_3_ and 100% CH_3_CN. The resulting peptide extracts were pooled, lyophilised in a vacuum centrifuge and stored at 4°C for subsequent protein identification. The protocol for protein identification was published by Lee et al. [32]. A nano LC/MS system consisting of a Surveyor HPLC system (Thermo Scientific, Waltham, MA, USA) and ESI-quadruple IT MS (LCQ Deca XP-Plus, Thermo Scientific, Waltham, MA) equipped with a nano-ESI source was used to perform the MS/MS experiments for protein identification. For desalting and concentration, 10 μl of digested sample was loaded by the auto sampler onto a C18 trap column (I.D 300 μm, length 5 mm, particle size 5 μm; DIONEX/LC Packings, Sunnyvale, CA) at a flow rate of 20 μl/min. To separate trapped peptides, the samples were back-flushed into a home-made microcapillary column (150 mm in length) packed with C18 resin (particle size 5 μm) in 75-μm silica tubing (8-μm id orifice) using mobile phases, A and B, composed of 0% and 80% of CH_3_CN containing 0.02% HCO_2_H and 0.5% CH_3_COOH, respectively.

The gradient initiated with 5% of the mobile phase B and 95% of the mobile phase A for 15 min. The mobile phase B was increased from 20% for 3 min, to 50% for 32 min, to 60% for 5 min, to 80% for 5 min, to 100% for 2 min and finally held at 100% for 8 min. The column was equilibrated and cleaned with 5% CH_3_CN for 10 min between running samples. The operating conditions for obtaining MS and MS/MS spectra were as follows: a capillary temperature of 220°C, an ESI voltage of 2.5 kV, and a collision energy setting at 35%. The three most abundant MS ions from MS were selected as peaks. Mass spectrometric analysis was included with the MS/MS analysis of the three most abundant ions from MS and 180 second dynamic exclusion. The Xcalibur data system (Therom Finnigan, USA) was used to control MS scan functions and HPLC solvent gradients. MS/MS spectra were searched against the proteins extracted with *Drosophila* from a non-redundant protein database at NCBI (ftp://ftp.ncbi.nlm.nih.gov/blast/db/FASTA/) using the SEQUEST (version 3.3.1, Thermo, USA) searching algorithm from the Bioworks (version 3.3.1, Thermo, USA) software package with the following parameters: a mass tolerance of 2.0 Da on the parent ion and 1.0 Da on the MS/MS, one missed cleavage per peptide allowed, and modifications of proteins were not considered. Mass peak lists were analysed for the *b* and *y* ions. The search results were filtered using the cross-correlation score (X_Corr_) and the normalised difference in cross-correlation scores (ΔC_n_). Matched peptide sequences had to pass the following thresholds: 1) the uniqueness scores of matches (ΔC_n_) were at least 0.1 and 2) minimum cross-correlation scores X_Corr_ of 1.9, 2.2, and 3.75 for charge states +1, +2, and +3, respectively. The search results were saved automatically. An SRF file including the merging of proteins, filter and sort settings, ratios and protein area/height values was used to select and sort peptide/spectrum matches passing this set of criteria. Proteins were identified using more than two peptides per spot.

### Anti-HSC3 western blot analysis

Ten adult fly heads were homogenised in ice-cold radio-immuno-precipitation assay (RIPA) buffer (150 mM NaCl, 1% IGEPAL CA-630, 0.5% Na-oxycholate, 0.1% SDS, 50 mM Tris, pH 8.0; Sigma-Aldrich, St. Louis, MO) with protease inhibitors (Halt™ protease inhibitor cocktail, Thermo Fisher Scientific, Rockford, IL). The homogenate was centrifuged at 14,000 *g* for 20 minutes at 4°C to remove cellular debris. The quantification of proteins in supernatants was accomplished using the BCA protein assay kit (Thermo Fisher Scientific).

For western blot analysis, 20.0 μg of total protein from each sample was separated on an 10% tri-glycine polyacrylamide SDS gel and then transferred to a nitrocellulose membrane (GE Healthcare Bio-Sciences AB, Uppsala, Sweden). The membranes were probed with rat-anti-HSC3 antibody (1:5,000; Babraham Bioscience Technologies Limited, Cambridge, UK) [32] and mouse monoclonal anti-α-tubulin antibody (1:2,000; Developmental Studies Hybridoma Bank, University of Iowa, Iowa City, IA) and were developed using the peroxidase-conjugated goat anti-rat IgG or goat anti-mouse-IgG (Thermo Fisher Scientific) and Supersignal West Pico (Thermo Fisher Scientific). The intensities of the bands were semi-automatically quantified using a wander tool and a histogram function in the Adobe Photoshop program (Adobe, San Jose, CA), as previously published [33]. Statistical analyses were performed with the Minitab software (Minitab Inc., State College, CA).

### Anti-HSP22 western blot analysis

HSP22 is a mitochondria resident chaperone [34]. The crude mitochondria pellet preparation method was modified from Miwa et al. [35] and Morrow et al. [36]. Briefly, flies were either not heat shocked or heat-shocked for 30 min at 37°C and allowed to recover for 4 hours at RT. Fly heads were grinded in 300 μl of ice-cold mitochondria homogenisation buffer (pH 7.4, 280 mM sucrose, 10 mM HEPES, 1 mM EDTA; Sigma-Aldrich) with protease inhibitors (Halt™ protease inhibitor cocktail, Thermo-Fisher Scientific). The homogenate was centrifuged at 750 g for 10 min at 4°C to remove cellular debris. To pellet crude mitochondria, the supernatant was centrifuged at 17,000 g for 10 min at 4°C. The pellet was re-suspended in 100 μl of ice-cold mitochondria homogenisation buffer. The BCA assay was then performed for protein quantification. A total of 2.98 μg of crude mitochondria samples was separated using 12% SDS-PAGE and then transferred to nitrocellulose membranes. The blots were probed with rabbit anti-HSP22 (1:5,000) [34] and developed with the peroxidase-conjugated goat anti-rabbit IgG (Thermo Fisher Scientific) and Supersignal West Pico (Thermo Fisher Scientific). Quantification and statistical analyses of band intensities were performed as described above.

### Anti-DTor antibody production and western blot analysis

His-tagged DTor recombinant peptides (residue 25–200) expressed in Esherichia coli M15 (Qiagen) were purified using Ni-NTA beads (Qiagen). After separated by SDS-PAGE, peptides were injected into rabbits for polyclonal antibody production.

Proteins were extracted from Tub-Gal4/+ and UAS-DTor/+;Tub-Gal4/+ larval body wall muscle preparation. After separated by a 10% SDS-PAGE, proteins were transferred to a nitrocellulose membrane (GE Healthcare Bio-Sciences AB). The membranes was probed with rat-anti-HSC3 antibody (1:5,000; Babraham Bioscience Technologies Limited) [32], mouse monoclonal anti-α-tubulin antibody (1:2,000; Developmental Studies Hybridoma Bank) and rabbit-anti-DTor antibodies (1:10,000) and were developed using the peroxidase-conjugated goat anti-rat IgG, goat anti-mouse-IgG, and goat anti-Rabbit-IgG (Thermo Fisher Scientific) and Supersignal West Pico (Thermo Fisher Scientific). Quantification and statistical analyses of band intensities were performed as described above.

### Quantitative real time PCR (qRT-PCR)

Total RNA was extracted from 30 heads of adult flies using Trizol**®** reagent (Invitrogen, Carlsbad, CA). The purity and integrity of the total RNA were determined according to the A_260_/A_280_ ratio and 28S rRNA/18S rRNA ratio, respectively. The A260/A280 ratio of the RNA samples used in this study was above 1.6, and the rRNA 28S/16S ratio was approximately 2:1 (data not shown). After treating with DNAse I (Promega, Madison, WI), complementary DNA (cDNA) was synthesised from 4 μg of total RNA using cDNA synthesis kits (Invitrogen). Quantitative real-time (qRT) PCR analysis was performed on a 7300 Real-Time PCR System (Applied Biosystems, Foster City, CA) using SYBR**®** Green PCR Master Mix (Applied Biosystems). The PCR conditions were 95°C for 10 min, followed by 40 cycles of 95°C for 15 s, 60°C for 20 s, and 72°C for 30 s for all target genes. The specificity of primer pairs (Additional file 1) used in this study was determined via dissociation curves and sequencing of amplified PCR fragments (data not shown). The PCR amplification efficiency of each primer pair was established using calibration curves (Additional file 2). Correlation coefficients (*r*^2^) and the slopes were > 0.98 and between −3.114 and −3.654, respectively, for all experiments (Additional file 2). The PCR efficiencies calculated for each primer pair using the equation = 10^-1/slope^-1 [37] were from 0.90126 to 1.09436 for all experiments (Additional file 2). Relative mRNA levels were adjusted with the internal control RP49 and calculated using the 2^-ΔΔCT^ method [37]. All reactions were performed in duplicate technically and in triplicate biologically.

### Anti-enhanced Green fluorescence Protein (eGFP) Western blot analysis

To examine the UPR activation *in vivo*, 10 day old adult brains of UAS-Xbp1-eGFP/Tub-Gal4, UAS-HTorA^WT^ and UAS-HTorA^ΔE^ /+;Tub-Gal4/UAS-Xbp1-eGFP were homogenised in RIPA buffer. A total of 15 μg of head protein extracts was separated using 10% SDS PAGE. Rabbit polyclonal anti-GFP antibody (sc-8334, Santa Cruz Biotechnology, Inc., Santa Cruz, CA) was used to detect the amount of Xbp1-eGFP. Anti-α-Tubulin antibody (DSHB) was used as a loading control. Quantification and statistical analyses of band intensities were performed as described above.

### Oxidative and ER stress sensitivity assay

To investigate the changes in oxidative and ER stress in *Drosophila*, a modified version of the oxidative and ER stress protocol from Meulener et al. [38] and Park et al. [33] was used. Briefly, each group of flies consisted of 5 males and 5 females that were 10 days old, which were transferred into a vial containing 1% agar, 5% sucrose with 20 mM paraquat (Sigma-Aldrich) or 1% H_2_O_2_ (Merck KGaA, Darmstadt, Germany) and kept at 25 ± 1°C and 60 ± 1% relative humidity. To induce ER stress, each group of flies was transferred to a vial containing 1% agar, 5% sucrose with 12 μM tunicamycin (Santa Cruz biotechnology, inc. Santa Cruz, CA), and 5 mM, 25 mM, or 100 mM dithiothreitol (DTT; Invitrogen, Carlsbad, CA). Flies were transferred to newly prepared tubes every other day. The number of dead flies was counted every 12 hours until 120 hours. Ten-independent tests were performed for each genotype. Two-sample t-tests were performed using Minitab software (Minitab). When the flies were treated with only 1% agar and 5% sucrose, there were no differences in survival rates between the HTorA^WT^- and HTorA^ΔE^-expressing flies (Additional file 3). A Kaplan-Meier survival analysis was performed using R (version 3.0.1. The R foundation for Statistical Computing) software.

### Autophagy inhibitor treatments

To inhibit autophagy, each group of flies consisting of 5 males and 5 females that were 10 days old were transferred into a vial containing 1% agar, 5% sucrose with 10 mM 3-methyladenine (3-MA;Sigma-Aldrich), 40 μM wortmanin (LC laboratory, Woburn, MA), and 40 μM LY294002 (LC laboratory) and kept at 25 ± 1°C and 60 ± 1% relative humidity as described previously [33]. The flies were transferred to newly prepared tubes every other day. The number of dead flies was counted every 12 hours. Ten-independent tests were performed for each genotype. A Kaplan-Meier survival analysis was performed using R (version 3.0.1. The R foundation for Statistical Computing) software.

### Density gradient sub-cellular fractionation

To obtain microsomes derived from endoplasmic reticulum in *Drosophila* brains, a modified version of the density gradient sub-cellular fractionation protocol from Xia et al. [39] and Tan et al. [40] was used. Briefly, one hundred adult fly heads were grinded and placed in 390 μl ice-cold isotonic homogenisation medium (0.25 M sucrose, 5 mM EDTA, 10 mM Tris–HCl, 50 mM KCl, pH7.5; Sigma-Aldrich) containing protease inhibitors (Halt™ protease inhibitor cocktail, Thermo-Fisher Scientific). The homogenate was centrifuged at 3000 g for 10 min at 4°C to remove cellular debris and nuclei. The amounts of proteins in the supernatants were measured using the BCA method, and equal amounts of proteins were used for further separations. The supernatant was then centrifuged in a S120AT2 rotor at 100,000 g for 1 hr at 4°C using a Himac CS 120EX (Hitachi, Japan). The resultant vesicle pellets were re-suspended in 1 ml of membrane suspension buffer (0.25 M sucrose, 50 mM KCl, 5 mM EDTA, 10 mM Tris–HCl, pH7.5; Sigma-Aldrich). Discontinuous Iodixanol gradients were generated using 60% Iodixanol (OptiPrep™, Axis-Shield PoC AS, Oslo, Norway) with dilution buffer (300 mM KCl, 30 mM EDTA, 60 mM Tris–HCl, pH 7.5) and set up in ultra-centrifuge tubes as follows: 40% Iodixanol, 100 μl; 35% Iodixanol, 150 μl; 30% Iodixanol, 200 μl; 25% Iodixanol, 350 μl; 20% Iodixanol, 500 μl; 10% Iodixanol, 450 μl; 5% Iodixanol, 250 μl; 2.5% Iodixanol, 100 μl). The re-suspended vesicle fractions were loaded on top of the gradients and centrifuged in a S100AT5 rotor at 340,000 g for 3 hr at 4°C using a Himac CS 120EX (Hitachi, Japan). The resulting gradient was harvested in 200 μl fractions. Ten μl from 12 fractions was separated using 8% SDS-PAGE and then transferred to nitrocellulose membranes. To identify ER fractions, the membranes were blotted with rat anti-HSC3 antibodies as described above.

### Protein extraction, SDS-PAGE and In-Gel digestion

One hundred μl of density gradient solutions from fraction Nos. 9 to 12 was boiled with SDS sample buffer for 10 min and concentrated using Vivaspin 500 (3,000 M.W. cut off; Satorius AG, Goettingen, Germany) before loading onto 10% SDS PAGE gels. The gels were stained with Coomassie brilliant blue. Each lane was split into 5 pieces that were digested individually with Trypsin-Gold (Promega) as described above. After incubation, digested peptides were extracted using an Oasis HLB extraction cartridge with C18 resin (Waters, MA, USA). Peptides in aqueous solution were loaded onto the columns washed with 0.5% Acetic acid/5% CH_3_CN and eluted in 0.5% Acetic acid/100% CH_3_CN. The peptide extracts were lyophilised in a vacuum centrifuge and stored at 4°C for subsequent identification and semi-quantification of proteins.

### Identification and semi-quantification of proteins present in HSC3 positive density gradient fractions using label-free proteomics

Previously published label-free proteomic methods [41,42] were modified and used. Lyophilised peptides were resuspended in 20 μl of 0.1% formic acid with 50 fmol/μl of MassPrep yeast enolase digestion standard (Waters) prior to LC/MS^E^ analysis. Each sample was analysed in three independent experimental runs with LC/MS^E^ using a nanoACQUITY ultra pressure liquid chromatography (UPLC) and a Synapt Q-Time of Flight (TOF) mass spectrometer equipped with a Nanolockspray ion source (Waters, Manchester, UK). A Mass PREP Digestion Standard (Protein Expression Mixture 1 & 2) (Waters) was run before and after the samples to monitor sensitivity and quantity. Two μl of sample was injected online onto a Waters Symmetry C18 trapping column (180 μm i.d. X 20 mm length with 5 μm particle size) at a flow rate of 10 μl/min in 0.1% formic acid for 5 min. Peptides were eluted off the precolumn and separated by in-line gradient elution onto a 75 μm i.d. X 200 mm length column packed with BEH130 C18 resin, 1.7 μm particle size, at a flow rate of 300 nL/min using a linear gradient from 3 to 45% B over 55 min (A; 0.1% formic acid in water, B; 0.1% formic acid in acetonitrile), followed by 25 min rinses at 90% B. The column was re-equilibrated with 3% B for 20 minutes prior to the next run. All column temperatures were maintained at 35°C. The mass accuracy was maintained during the run using Nanolockspray of the peptide [glu1]-fibrinopeptide B delivered through the auxiliary pump of NanoACQUITY at a concentration of 400 fmol/μL and a flow rate of 500 nL/min. The peptides were analysed in positive ion mode and were operated in v-mode with a typical resolving power of 10,000 fwhm. Prior to the analyses, the TOF analyser was calibrated using [glu1]-fibrinopeptide B fragments that were obtained using a collision energy of 30 eV and over the mass range 50–1990 m/z. The Q-TOF was operated in the LC/MS^E^ mode of acquisition. The MS^E^ mode was programmed to acquire data with a proprietary dual exact mass protocol. The two acquisition functions were rapidly alternated between low and elevated collision energies. One second scans of low and elevated high collision energy resulted in the acquisition of a time-resolved global protein expression dataset containing two data functions; the first was composed exclusively of conventional, low energy MS spectra (intact peptide ions), and the second was composed of mass spectra acquired at an elevated high collision energy (peptide product ions). Low energy data collections were performed at a constant collision energy of 4 eV, and high collision energy acquisitions were performed using a 15–40 eV ramping. The [glu1]-fibrinopeptide at a concentration of 400 fmol/μL (m/z 785.8426) was infused via the nanolockspray ion source at a flow rate of 500 nL/min and sampled every 30 s as an external mass calibrant. For each injection, the mass spectrometer acquired data from 0 to 90 min. The Water Protein Expression System Informatics incorporated in ProteinLynx Global SERVER version 2.4 (PLGS 2.4) was used to process all raw data files. Each processed file was then searched against the *Drosophila melanogaster* protein database obtained from UniProt (http://www.uniprot.org/uniprot/?query=taxonomy:7227) with *Saccharomyces cerevisiae* enolase (P00924) appended. Protein identifications from the low/high collision spectra for each sample were processed using a hierarchical approach where detection of at least 3 fragment ion matches per peptide, 7 fragment ion matches per protein and 2 peptide matches per protein were required. The fixed and variable modifications considered were carbamidomethylation of cysteine residues and oxidation of methionine, respectively, with one allowable missed cleavage. All proteins identified with high confidence and with more than two peptides were considered for protein quantification. In relative protein quantitative analyses, multiple normalised global expression datasets were compared and contrasted. Exact mass retention time (EMRT) signatures enabled all detected proteins to be uniquely identified. The EMRT signatures of any two global expression datasets to be compared were matched. The normalised intensity of each EMRT signature was indicative of the abundance of a specific tryptic peptide. Each protein in a global expression dataset was represented by multiple peptides, so the relative quantitation of any protein was calculated by comparing an average and relative protein fold-change of multiple matched EMRT signatures. We considered significantly regulated proteins with a likelihood of quantification smaller than 0.05. The entire data set of differentially expressed proteins was further filtered by considering the identified peptides that replicated two out of three technical instrument replicates with an over 80 protein probability score (Additional file 4). The label-free semi-quantification results of density gradient fractions were summarised in Additional file 5.

### Life span analysis of the HTorA^ΔE^ flies with down-regulated UPR sensors and inducers in the neuronal system

The expression of *hsc3, xbp1, ATF6* and *PEK* in the neuronal systems of the flies was down-regulated by crossing RNAi construct flies with pan-neuronal Gal4 drivers, C155-Gal4. The levels of transcripts of each gene down-regulated by its RNAi flies were confirmed using qRT-PCR (Additional file 6). Among 3 lines of the *hsc3*-RNAi flies tested, only one line that gave rise to the adult flies when crossed with C155-Gal4 driver flies was used to test the consequences of down-regulated *hsc3* at the neuronal systems with (C155/+;*hsc3*-RNAi/+) or without HTorA^ΔE^ (C155/+;UAS-HTorA^ΔE^/*hsc3*-RNAi). The *xbp1*-RNAi and the *xbp1*^*K13803*^/CyO flies previously characterised [43] were used in this study to examine the consequences of a reduced amount of xbp1 in fly brains with or without HTorA^ΔE^. The *ATF6*-RNAi and the *PEK*-RNAi flies were crossed with the pan-neuronal Gal4 flies with or without HTorA^ΔE^-expression, and then the consequences were examined. The numbers of live flies in 12 ~ 15 vials of each genotype were counted every 3 ~ 4 days. Only female flies raised at 25°C were used for this analysis. A Kaplan-Meier survival analysis was performed using R (version 3.0.1. The R foundation for Statistical Computing) software.

## Results

### Six proteins in 7 spots were differentially expressed in HTorA^ΔE^-expressing fly brains

**Figure 1:**
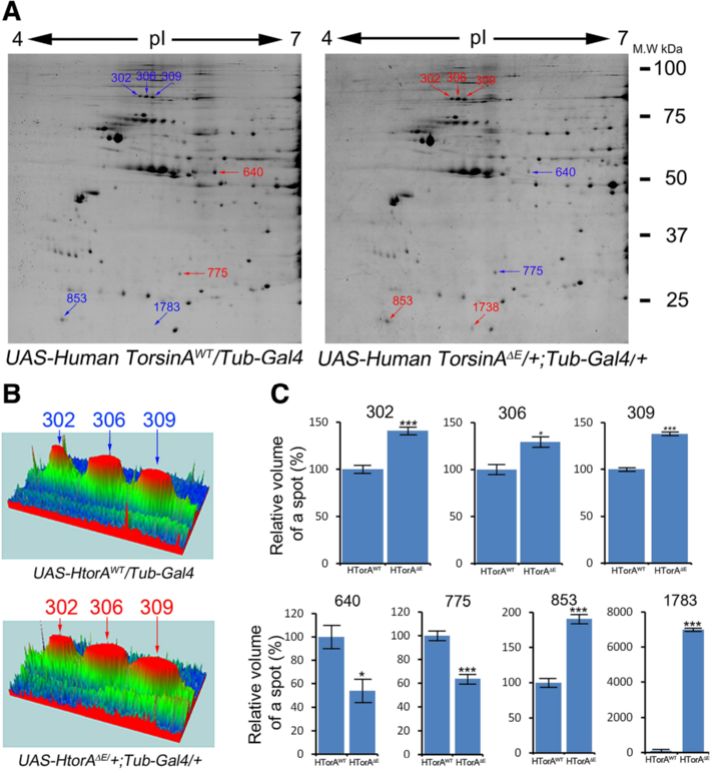
A representative comparison of protein expression patterns between fly brains expressing HtorA^WT^ and HtorA^ΔE^ using 2-DE. **(A)** Differentially expressed protein spots of the HTorA^WT^- and the HTorA^ΔE^ –expressing flies were labeled with Coomasie brilliant blue staining. Blue colors indicate lower amounts of proteins, and red colors represent significantly more proteins. **(B)** Representative 3D-views of 3 differentially expressed spots. **(C)** Quantification results of differentially expressed spots. * = p < 0.05, *** = p < 0.001.

Figure 1A shows representative 2-dimensional electrophoresis (DE) images of soluble proteins extracted from adult fly brains expressing human TorsinA (HTorA)^WT^ and HTorA^ΔE^. The normalised volumes of spot 302, 306, 309, 853 and 1783 were significantly increased in the HTorA^ΔE^-expressing brains, whereas the normalised volumes of spots 640 and 775 were significantly decreased in the HTorA^ΔE^-expressing brains (Figure 1). Even though proteins in spots 302, 306 and 309 had different isoelectric points, similar size-dependent migration patterns suggested that the major proteins in these spots might have been the same proteins with different post-transcriptional modifications (Figure 1B and C). Indeed, Heat shock protein cognate 70–3 (HSC3) was commonly identified in spots 302, 306, and 309 (Figure 1C, Table 1, and Additional file 7). In addition, Transferrin 1 (Tsf1) was identified in spots 302 and 309 (Figure 1B, Table 1, and Additional file 7). Cuticular protein 72Ec (Cpr72), lethal (2) 37Cc, Sarcoplasmic calcium binding protein 1 (Scp1), and Heat shock protein 22 (HSP22) were identified in spots 640, 775, 853, and 1783, respectively (Table 1, Additional file 7).

**Table 1:** **Summary of identified proteins from spots with significantly altered volumes**

**2D spot No.**	**Gene bank Accession No.**	**Gene Name**	**Sequence Coverage (%)**	**Unique peptide detected**	**Predicted M.W. (kDa)**	**Predicted pI (pH)**
302	AAN09299.1	Heat shock protein	30.48	12	72.27	4.99
		cognate 3	6.71	3	71.83	7.07
		(HSC3)				
	AAF48831.1	Transferrin 1 (Tsf1)				
306	AAN09299.1	HSC 3	22.26	10	72.27	4.99
309	AAN09299.1	Heat shock protein	18.60	7	72.27	4.99
	AAF48831.1	cognate 3	4.52	2	71.83	7.07
		Tsf 1				
640	AAF49476.1	Cuticular protein 72Ec	11.19	3	50.58	5.15
775	AAN11026.1	lethal (2) 37Cc	25.72	5	28.3	5.80
853	EAA46049.1	Sarcoplasmic calcium-binding protein 1	39.68	5	21.65	4.50
1783	AAN11962.1	Heat shock protein 22	25.28	3	19.77	4.95
		(HSP22)				

### Significantly increased HSC3 and HSP22 proteins in HTorA^ΔE^-expressing brains

**Figure 2:**
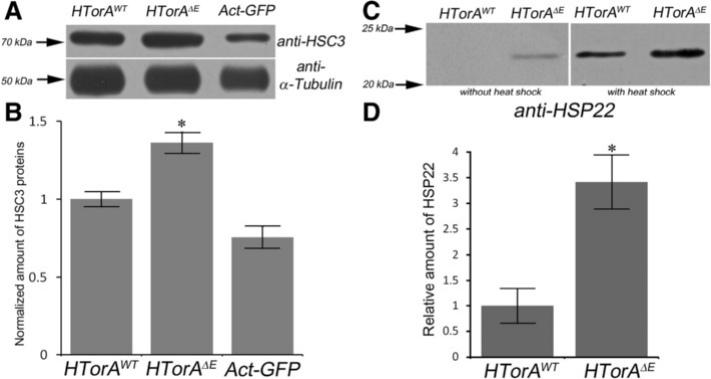
The HTorA^ΔE^-expressing flies had significantly increased HSC3 and HSP22 in their brains. **(A and B)** When the amounts of HSC3 were normalised with α-Tubulin as a loading control, the HTorA^ΔE^-expressing brains had 1.4-fold more HSC3 than those of the HTorA^WT^-expressing brains. However, Actin-enhanced Green fluorescence protein (Act-eGFP) expressing flies showed similar expression levels of HSC3. **(C and D)** In normal condition, HSP22 was detected from only the crude mitochondrial fraction of the HTorA^ΔE^-expressing brains and it was 3.4-fold more abundant in the crude mitochondrial fraction of the HTorA^ΔE^ -expressing brains than that of the HTorA^WT^-expressing brains before or following a heat shock. * = p < 0.05.

HTorA belongs to the AAA+ ATPase family and localises to the lumen of the endoplasmic reticulum (ER) [16]. The consequence of a ΔE mutation in HTorA was a preferential localisation of the HTorA aggregates at the nuclear envelope (NE) and in the ER in diverse animal and cellular models [29,44,45]. We further verified the increased expression of HSC3 and HSP22 in the HTorA^ΔE^-expressing brains compared to the HTorA^WT^-expressing brains by performing western blot analysis using rat anti-HSC3 and rabbit anti-HSP22 antibodies. The HSC3 proteins in the HTorA^ΔE^-expressing brains were increased 1.4-fold compared to the levels expressed in the HTorA^WT^-expressing brains. However, HSC3 proteins in Act-eGFP expressing brains were similar to those of HTorA^WT^-expressing brains (Figure 2A and B). We also further tested whether expression of Drosophila Torsin (DTor) induced any change in HSC3 expression. Even DTor proteins in DTor overexpressing brains were increased 3.5-fold compared to the levels of Tub-Gal4/+ control flies, there was no change in HSC3 expression (Additional file 8). In addition, HSP22 proteins were detected from only the HTorA^ΔE^ flies in normal condition (Figure 2C), and a 3.4-fold increase of HSP22 was observed in the HTorA^ΔE^-expressing brains compared to the HTorA^WT^-expressing brains following heat shock (Figure 2C and D). These data confirmed that the amount of HSC3 and HSP22 were increased in the HTorA^ΔE^-expressing brains.

### Transcripts of HSC3 and HSP22 were increased in HTorA^ΔE^-expressing brains

**Figure 3:**
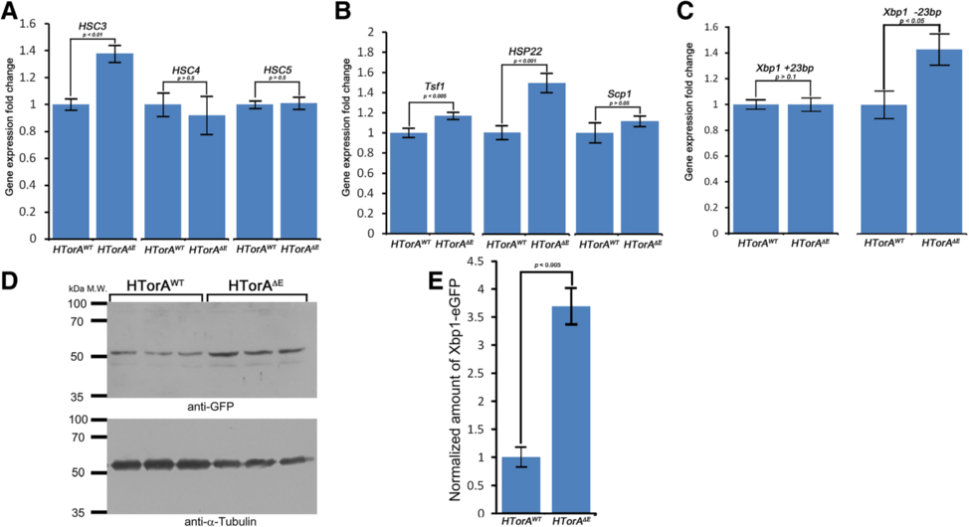
Transcripts of HSC3, HSP22, Tsf1 and IRE1-dependent spliced Xbp1 and Xbp1-eGFP signals were significantly increased in the HTorA^ΔE^-expressing brains. Quantitative-RT-PCR results of genes encoding proteins displaying the largest alterations in expression in the 2-DE analysis. **(A)** Only the transcripts of *heat shock protein cognate 3* (*hsc3*) but not those of *hsc4* and *hsc5* were significantly increased in the HTorA^ΔE^ flies. **(B)** The transcripts of *transferrin1* (*tsf1*), *heat shock protein 22* (*hsp22*), and *sarcoplasimc calcium binding protein* (*scp1*) were not changed. **(C)** The amount of the IRE1-dependent spliced form of Xbp1 mRNA (Xbp1 -23 bp) was increased in the HTorA^ΔE^ brains. **(D)** Compared with HTorA^WT^, expression of HTorA^ΔE^ induced increased expression of Xbp1-eGFP in *Drosophila* heads. α-Tubulin was used as a loading control. **(E)** The normalised amount of the Xbp1-eGFP band in the HTorA^ΔE^ expressing brains was 3.68-fold higher than that in the HTorA^WT^-expressing brains.

On the basis of the proteomic results for hsc3 and hsp22, we next checked their transcript levels compared to those of controls by performing quantitative real-time PCR (qRT-PCR). The transcripts of *hsc3*, *tsf1* and *hsp22* in the HTorA^ΔE^-expressing fly brains were significantly higher than those in the HTorA^WT^-expressing fly brains, but there was no difference in transcript levels of *scp1* between the HTorA^WT^- and the HTorA^ΔE^-expressing brains (Figure 3A and B). Because *hsc3* has two very closely related genes (*hsc4* and *hsc5)* in the fly genome, we confirmed that there were no differences in the levels of expression of *hsc4* and *hsc5* transcripts between the HTorA^WT^- or the HTorA^ΔE^-expressing brains.

### Increased IRE1-dependent spliced x-box binding protein 1 (xbp1) mRNA in the HTorA^ΔE^-expressing brains

Previous studies have shown that the transcripts of *hsc3* and *hsp22* are increased when flies are exposed to ER stress that activates the UPR [31] and oxidative stress [46], respectively. To determine whether the UPR in the HTorA^ΔE^-expressing flies was induced, the levels of unspliced and IRE1-dependent spliced forms of x-box binding protein 1 (*xbp1*) mRNA were examined using performing qRT-PCR. Previous studies in *Drosophila* have shown that the levels of an IRE1-dependent spliced form of *xbp1* mRNA are increased upon activation of the UPR [31,47]. The levels of unspliced *xbp1* mRNA were similar between the HTorA^WT^- and the HTorA^ΔE^-expressing brains. In contrast, the level of spliced *xbp1* mRNA was significantly increased in the HTorA^ΔE^-expressing brains compared with those of the HTorA^WT^-expressing flies (Figure 3C). These results suggest that HTorA^ΔE^ initiates the UPR in the ER.

To further confirm that the IRE1-dependent splicing of *xbp1* mRNA was induced by HTorA^ΔE^, we used a xbp1-eGFP reporter that allowed eGFP to be expressed only when a 23 bp intron was spliced out of the xbp1-eGFP construct by IRE1 in the ER [31]. Compared with protein extracts of the brains of the control flies (UAS-xbp1-eGFP/Tub-Gal4, UAS-HTorA^WT^), the brains of the HTorA^ΔE^-expressing flies (UAS-HTorA^ΔE^/+; Tub-Gal4/UAS-xbp1-eGFP) showed significantly increased amounts of Xbp1-eGFP signals that were detected using anti-GFP antibodies (Figure 3D and E). These results show that HTorA^ΔE^ activates the UPR through IRE1-dependent splicing of Xbp1 mRNA.

### Increased oxidative and ER stresses in the HTorA^ΔE^-expressing flies

**Figure 4:**
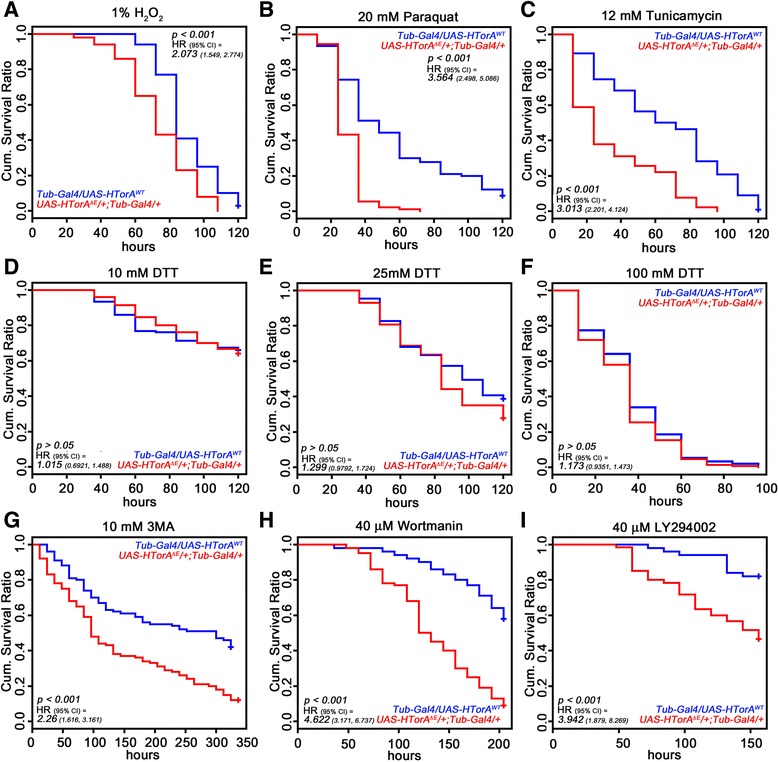
Increased sensitivities to oxidative and ER stressors and autophagy inhibitors in the HTorA^ΔE^ flies. **(A)** The hazard ratio (HR) of the HTorA^ΔE^ expressing flies reared on fly food containing 1% H_2_O_2_ was 2.073 times increased compared to those of the HTorA^WT^ expressing flies. **(B)** The HTorA^ΔE^-expressing flies also showed 3.564 times increased HR to 20 mM paraquat induced oxidative stress. **(C)** When ER stress was induced using 12 mM tunicamycin, the HR of the HTorA^ΔE^-expressing flies was 3.013 times increased compared with the HTorA^WT^-expressing flies. **(D-F)** When a protein disulfide bond reducing compound DTT was applied with three different concentrations, including 5 mM **(D)**, 25 mM **(E)**, and 100 mM **(F)**, there was no difference in HR between the HTorA^WT^ and the HTorA^ΔE^-expressing flies. **(G-I)** The HTorA^ΔE^ flies were vulnerable to autophagy inhibitors, 10 mM 3-MA **(G)**, 40 μM Wortmannin **(H)**, and 40 μM LY294002 **(I)** compared with the HTorA^WT^ flies. HR = hazard ratio, 95% CI = 95% confident intervals of hazard ratio.

The altered transcription and protein expression of HSC3 and HSP22 in the HTorA^ΔE^-expressing fly brains suggested that the HTorA^ΔE^-expressing flies might have altered susceptibilities to oxidative and/or ER stress. Previous studies have shown that the susceptibility of flies to oxidative stress can be determined by treating them with H_2_O_2_ or paraquat [38]. When flies were reared on foods containing 1% H_2_O_2_, the HTorA^ΔE^-expressing flies exhibited significantly more susceptibility to H_2_O_2_-induced oxidative stress than the HTorA^WT^-expressing flies (Figure 4A). The hazard ratio (HR) of the HTorA^ΔE^-expressing flies was 2.073 compared with the HTorA^WT^-expressing flies. In addition, the HR of the HtorA^ΔE^-expressing flies was 3.564 compared with the HTorA^WT^-expressing flies when exposed to 20 mM paraquat (Figure 4B).

The HR of the HTorA^ΔE^-expressing flies to tunicamycin induced ER stress was 3.013 compared with the HTorA^WT^-expressing flies (Figure 4C). However, there was no difference in dithiothreitol (DTT) sensitivity between the HTorA^WT^- and the HTorA^ΔE^-expressing flies when the flies were treated with 5 mM, 25 mM or 100 mM DTT (Figure 4D-F). Because tunicamycin and DTT have been known to activate UPR signalling by a different mechanism, these results suggest that HTorA^ΔE^ may induce the activation of specific pathways in the UPR.

### HTorA^ΔE^-expressing flies showed increased sensitivities to autophagy inhibitors

In a previous study, we showed that protein aggregates induced by HTorA^ΔE^ localised to the nuclear membrane, mitochondria, synapses, single membrane lysosomes and double membrane autophagosomes [29]. Because mutations that are known to cause defects in autophagy are related to various neurological disorders [11,48], we further investigated whether HTorA^ΔE^ flies showed altered sensitivities to 3 different autophagy inhibitors (Figure 4 G, H, and I). These results suggested that formations of autophagosomes in HTorA^ΔE^ flies may be involved in protecting flies from HTorA^ΔE^ induced toxicities.

### Altered transcript levels of Atf6, Calreticulin, *Drosophila* glucose regulated protein 170 (dGRP170), Activating transcription factor 4 (Atf4), and components of the ERAD pathway in the HTorA^ΔE^-expressing brains

**Figure 5:**
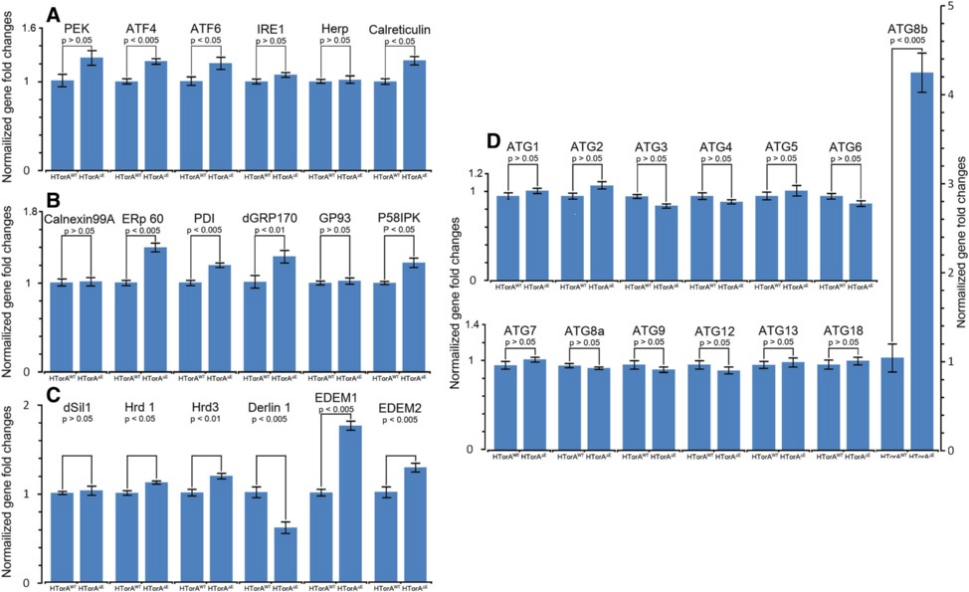
Transcripts of some ER stress sensors, chaperones, components of ERAD, and ATG8b were significantly altered in the HTorA^ΔE^-expressing brains. The amount of *ATF4, ATF6, calreticulin* **(A)**, *ERp60, PDI, dGRP170, P58IPK* **(B)**, *Hrd1, Hrd3, Derlin-1, EDEM1, EDEM2* **(C)** and *ATG8b* **(D)** transcripts in the HTorA^ΔE^-expressing brains was significantly different from those of the HTorA^WT^-expressing brains.

To further investigate the molecular and cellular mechanisms underlying UPR activation in the HTorA^ΔE^-expressing brains, the levels of transcripts of three UPR inducers and ER-stress (ERSE) and UPR target genes (UPRE) [7,49] were quantified and compared with those of the HTorA^WT^-expressing brains by performing qRT-PCR (Additional files 1 and 2). Of the three UPR inducers, the transcript level of Atf6 was significantly increased in the HTorA^ΔE^-expressing flies (Figure 5A). The expression of the stress-responsive activating transcription factor-4 (Atf4), a downstream regulator of the UPR genes that is activated by *Drosophila* PERK (PEK), was significantly increased (Figure 5A). In addition, the transcription levels of Calreticulin, ERp60, PDI, *Drosophila* homologue of GRP170 (dGRP170), and P58IPK were significantly different, whereas those of Calnexin 99A, GP93 (*Drosophila* homologue of GRP94), and *Drosophila* homologue of Sil1 (dSil1) were the same between the two fly groups (Figure 5A-C). Among the ERAD components tested, the transcripts of Hrd1, Hrd3, Derlin-1, EDEM1, and EDEM2 were also significantly different (Figure 5C).

Because the HTorA^ΔE^ flies showed significantly increased susceptibilities to autophagy inhibitors (Figure 4 G-4I), we also examined any change in the expression of autophagy-related genes (Atgs) in the HTorA^ΔE^ flies. Among 13 Atgs tested, only Atg8b was found to have a significant difference in expression (Figure 5D and E). Taken together, the qRT-PCR results indicated that the three UPR inducers in the ER were activated by HTorA^ΔE^ and that the expression of certain ER chaperones, nucleotide exchange factors, ERAD components and ATG8b was also induced.

### HSC3 positive density gradient fractions of HTorA^ΔE^ and HTorA^WT^ microsomes harboured different protein profiles

**Figure 6:**
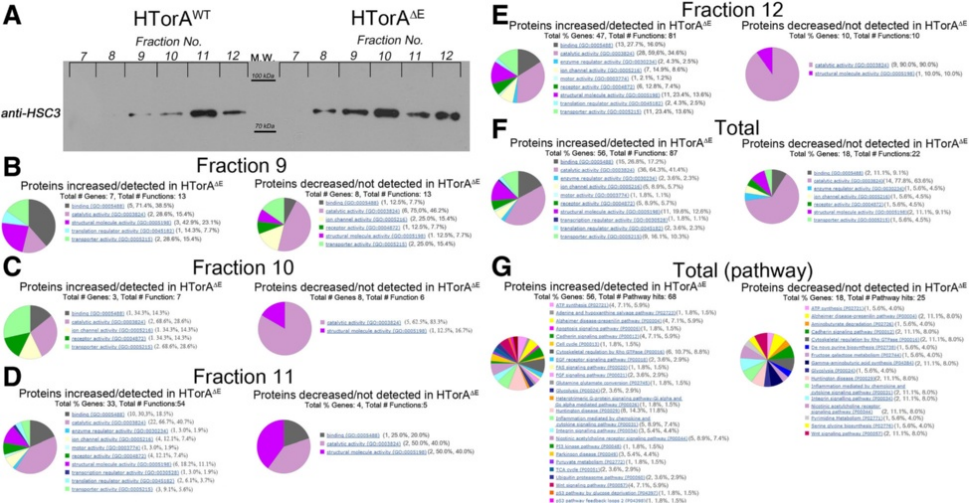
The distribution patterns of HSC3 in Optiprep density gradient factions acquired from the HTorA^ΔE^-expressing brains were different from those of the HTorA^WT^-expressing brains. **(A)** HSC3 was present in fraction Nos. 9–12 in the HTorA^WT^-expressing brains, with the strongest signals in fraction No. 11. However, HSC3 was detected from fraction Nos. 8–12 in the HTorA^ΔE^-expressing brains with the strongest signals found in fraction No. 10. **(B ~ E)** Proteins identified from fraction 9 **(B)**, 10**(C)**, 11**(D)**, and 12**(E)** from the HTorA^ΔE^-expressing brains were different from those form the HTorA^WT^-expressing brains. **(F)** When all proteins identified from Faction 9 to 12 were pooled, fractions from HTorA^ΔE^-expressing brains had 56 genes that were increased or unique, whereas 18 genes were increased or unique from the HTorA^WT^-expressing brains. Gene ontology profiles for functions **(F)** and pathways **(G)** were displayed, respectively.

In addition to their functions as ER stress chaperones [49,50], Bip and its homologues are involved in many molecular chaperone activities in the ER, such as importing newly synthesised proteins across the ER membrane, increasing the possibility of the correct protein folding, assembly and oligomerisation of the newly synthesised proteins, and controlling Ca^2+^ homeostasis [51,52]. Because several lines of evidence in this study suggested that the HTorA^ΔE^ expression in fly brains may activate the UPR in the ER and perturb ER homeostasis, we reasoned that the biophysical and biochemical properties of the ER in the HTorA^ΔE^ flies might be different than those in the HTorA^WT^ flies. We directly addressed this question by obtaining ER microsomes via density gradient ultra-centrifugation followed by anti-HSC3 western blot analysis and profiling the constituent proteins in those fractions via LC/MS^E^ proteomic analysis. Interestingly, the fraction Nos. 9–12 obtained from HTorA^WT^ contained HSC3, whereas the fraction Nos. 8–12 from HTorA^ΔE^ were HSC3 positive (Figure 6A). In addition, the fractions that had the most abundant HSC3 immunoreactivity were No. 11 in the HTorA^WT^-expressing brains versus No. 10 in the HTorA^ΔE^-expressing brains. Because sedimentation equilibrium density is known to be determined by the sizes, densities, and molecular weights of the proteins comprising macromolecular complexes, differences in the intensities of HSC3 signals in the microsome fractions of the HTorA^ΔE^- and the HTorA^WT^-expressing brains suggested that the diversity and quantity of the proteins in the HSC3 positive microsomes might differ from each other.

Indeed, 35 proteins in fractions No. 9, 46 proteins in fractions No. 10, 109 proteins in fractions No. 11, and 160 proteins in fractions No. 12 from the HTorA^ΔE^ and the HTorA^WT^ microsomes were identified using LC/MS^E^ (Additional file 4). Eight, 4, 41, and 58 proteins in fractions No. 9, 10, 11 and 12, respectively, were increased or detected in the HTorA^ΔE^ microsomes, and 9, 10, 6, and 14 proteins in fractions No. 9, 10, 11 and 12, respectively, were decreased or not detected in the HTorA^ΔE^ microsomes. Greater numbers and types of proteins were present in the HTorA^ΔE^ microsomes than in the HTorA^WT^, suggesting that HTorA^ΔE^ in the ER may impair the trafficking and secretion of proteins resulting in ER overloads, which is another known ER stressor.

The quantitatively and qualitatively altered proteins in each fraction were divided into groups according to Gene Ontology (GO) molecular functions using Panther version 7 (http://pantherdb.org) [53] and AmiGO, the Gene ontology (http://amigo.geneontology.org/amigo) [54] (Figure 6 B-G). Of the proteins that were increased or unique to the HTorA^ΔE^-expressing brains, 71.4% of the annotated proteins in fraction 9 had binding activity (GO:0005488), whereas 68.6%, 66.7% and 59.6% of the annotated proteins in fractions No. 10, 11, and 12, respectively, had catalytic activity (GO:0003824). Other proteins had structural molecule activity (GO:0005198), translation regulator activity (GO:0045182), transporter activity (GO:0005215), ion channel activity (GO:0005216), receptor activity (GO:0004872), enzyme regulator activity (GO:003234), motor activity (GO:0003774), and transcription regulator activity (GO:0030528) (Figure 6 B-E and Additional file 9). Furthermore, of the proteins that were decreased or not present in the HTorA^ΔE^-expressing brains, 75.0%, 62.5%, 50.0% and 90.0% of the annotated proteins from fractions No. 9, 10, 11, and 12, respectively, had catalytic activity (GO:0003824). In addition, the proteins with structural molecule activity (GO:0005198) were consistently decreased in all lanes. Other decreased proteins had binding activity (GO:0005488), ion channel activity (GO0005216), receptor activity (GO:0004872), and transporter activity (GO:0005215) (Figure 6B-E and Additional file 10). When all proteins from fractions No. 9 to 12 were combined for comparison, 68 proteins were increased or unique to HTorA^ΔE^ microsomes. Among 56 annotated proteins, 64.3%, 26.8%, and 19.6% had catalytic activity (GO:0003824), binding activity (GO:0005488) and structural molecule activity (GO:0005198), respectively. Of the 21 proteins decreased or not present in the HTorA^ΔE^ microsomes, 77.8% and 11.1% of the 18 annotated proteins had catalytic activity (GO0003824) and binding activity (GO:0005198), respectively (Figure 6F).

The dysregulated proteins in the ER microsomes were further characterised by their association with 165 regulatory and metabolic pathways in the Panther Pathway 3.01 [53]. The following pathways had more than 5 genes associated with them when up-/down-regulated genes were considered simultaneously: the Huntington’s disease pathway (P00029), the cytoskeletal regulation by Rho GTPase pathway (P00016), the inflammation mediated by chemokine and cytokine signalling pathway (P00031), the nicotinic acetylcholine receptor signaling pathway (P00044), the Alzheimer’s disease-presenilin pathway (P00004), the Cadherin signaling pathway (P00012), the Wnt signaling pathway (P00057) and the ATP synthesis pathway (P02721) (Figure 6 G). These profiling results indicated that HTorA^ΔE^ in the ER involved defects in the synthesis, modification, folding, assembly and trafficking of secretory and membrane proteins that translocate to the cell surface or to intracellular organelles.

### Down-regulation of HSC3, Xbp1, ATF6 and Pek in the HTorA^ΔE^ flies induced a significantly earlier death

**Figure 7:**
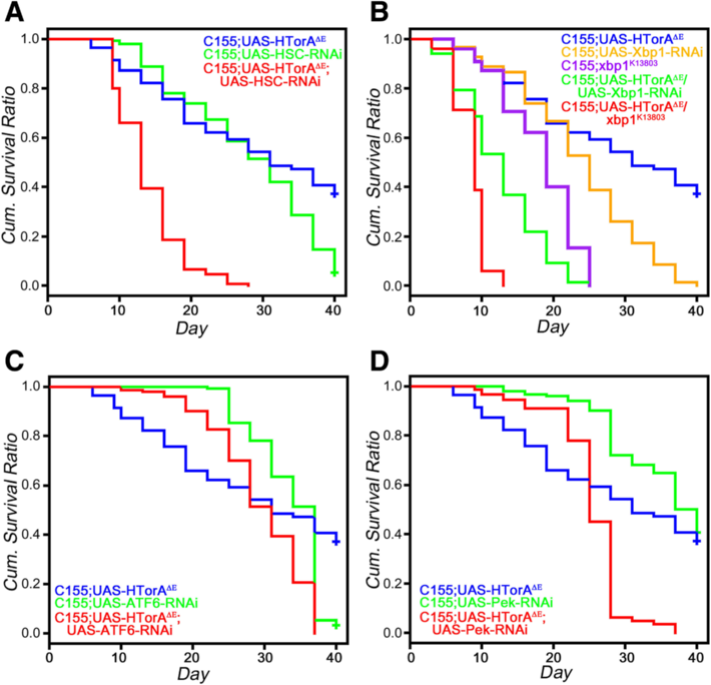
Down-regulation of *hsc3, xbp1, ATF6* and *Pek* induced early death of the HTorA^ΔE^ flies. **(A)** When *hsc3* expression was down-regulated by *hsc*-RNAi, it resulted in the induction of early death for the HTorA^ΔE^ flies. **(B)** Down-regulation of Xbp1 by mutant chromosome, *xbp1* ^*K13803*^ or *xbp1* RNAi resulted in significantly earlier deaths among the HTorA^ΔE^ flies. **(C)** Decreased ATF6 in the HTorA^ΔE^ flies induced increased the HR of the HTorA^ΔE^ flies. **(D)** Down-regulated Pek in the HTorA^ΔE^ fly brains significantly increased the HR of the HTorA^ΔE^ flies.

To understand the physiological consequences of UPR activation in the HTorA^ΔE^ flies, we genetically down-regulated the expression of *hsc3, xbp1, ATF6* or *Pek* in the HTorA^ΔE^ flies using RNAi flies for each gene (Additional file 6). When the expression of *hsc3* was down-regulated in the HTorA^ΔE^ flies by RNAi constructs, its HR was 6.579 or 8.135 compared with C155/+; UAS-HTorA^ΔΕ^/+ or C155/+; UAS-HSC3-RNAi flies, respectively (Figure 7A and Table 2). We further examined the consequences of the down-regulated expression of *xbp1* by expressing *xbp1*-RNAi constructs or using the *xbp1* mutant chromosome *xbp1*^*K13803*^ in the HTorA^ΔE^ flies. The down-regulation of *xbp1* expression by *xbp1*-RNAi constructs or mutant chromosome *xbp1*^*K13803*^ in C155/+; UAS-HTorA^ΔΕ^/+ significantly increased the HR to 6.796 or 21.23 compared with C155/+; UAS-HTorA^ΔΕ^/+, respectively (Figure 7B and Table 2). The HR of those flies were 5.871 or 15.42 when compared with C155/+; UAS-Xbp1-RNAi or C155/+; *xbp1*^*K13803*^ flies, respectively (Figure 7B and Table 2). The effect of down-regulation of ATF6 by RNAi in HTor1A^ΔE^ flies was less pronounced but also resulted in a significant induction of early death. The HR of C155/+; UAS-HTorA^ΔΕ^/UAS-ATF6-RNAi flies were 2.222 and 1.976 compared with C155/+;UAS-HTorA^ΔΕ^/+ or C155/+; UAS-ATF6-RNAi/+ flies (Figure 7C and Table 2). In addition, the down-regulation of PEK by *PEK*-RNAi in C155/+;UAS-HTorA^ΔΕ^ flies induced a significantly earlier death of the flies. The HR of C155/+;UAS-HTorA^ΔE^/UAS-Pek-RNAi were 2.914 or 7.408 compared with C155/+;UAS-HTorA^ΔE^/+ or C155/+;UAS-Pek-RNAi (Figure 7D and Table 2).

**Table 2:** **The summary of Kaplan-Meyer survival analysis with altered expression of UPR sensor and inducers in HTorA** ^**ΔE**^ **-expressing flies**

**Genotype**			***p*** **-value** ^**1**^	**Hazard ratio (95% CI)** ^**2**^
**Control**		**Trial**		
A	C155-Gal4/+;UAS-HTor1A^ΔE^/+	C155-Gal4/+; UAS-HSC3-RNAi/UAS-HTor1A^ΔE^	<0.001	6.579 (4.726, 9.159)
	C155-Gal4/+;UAS-HSC3-RNAi/+		<0.001	8.135 (5.87, 11.27)
B	C155-Gal4/+;UAS-HTor1A^ΔE^/+	C155-Gal4/+;UAS-HTor1A^ΔΕ^/UAS-Xbp1-RNAi	<0.001	6.796 (4.865, 9.492)
		C155-Gal4/+;UAS-HTor1A^ΔΕ^/xbp1^K13803^	<0.001	21.23 (13.19, 34.17)
	C155-Gal4/+; UAS-Xbp1-RNAi	C155-Gal4/+;UAS-HTor1A^ΔΕ^/UAS-Xbp1-RNAi	<0.001	5.871 (4,351, 7.924)
	C155-Gal4/+; xbp1^K13803^/+	C155-Gal4/+;UAS-HTor1A^ΔΕ^/xbp1^K13803^	<0.001	15.42 (10.38, 22.9)
C	C155-Gal4/+;UAS-HTor1A^ΔE^/+	C155-Gal4/+;UAS-HTor1A^ΔE^/UAS-ATF6-RNAi	<0.001	2.222 (1.672, 2.954)
	C155-Gal4/+;UAS-ATF6-RNAi/+		<0.001	1.976 (1.562, 2.499)
D	C155-Gal4/+;UAS-HTor1A^ΔE^/+	C155-Gal4/+;UAS-HTor1A^ΔE^/UAS-Pek-RNAi	<0.001	2.914 (2.158, 3.934)
	C155-Gal4/+;UAS-Pek-RNAi/+		<0.001	7.408 (5.368, 10.22)

^1^ *p*-values were calculated using the log-rank test.

^2^Hazard ratios and its 95% confidential intervals (CI) were estimated using cox-regression.

## Discussion

The aim of this study was to elucidate the molecular and cellular consequences of HTorA^ΔE^ in *Drosophila* brains to gain insight into the pathogenesis of DYT1 dystonia. The most interesting finding was that the expression of the ER molecular chaperone HSC3 and a mitochondria specific chaperone HSP22 were significantly increased in the HTorA^ΔE^-expressing brains obtained from unbiased 2-DE analysis (Figure 1, 2, and Table 1). Those alterations were further verified using western blot and qRT-PCR analyses (Figure 3 and 4).

### Three axes of UPR signaling in the HTorA^ΔE^ brains may be activated

**Figure 8:**
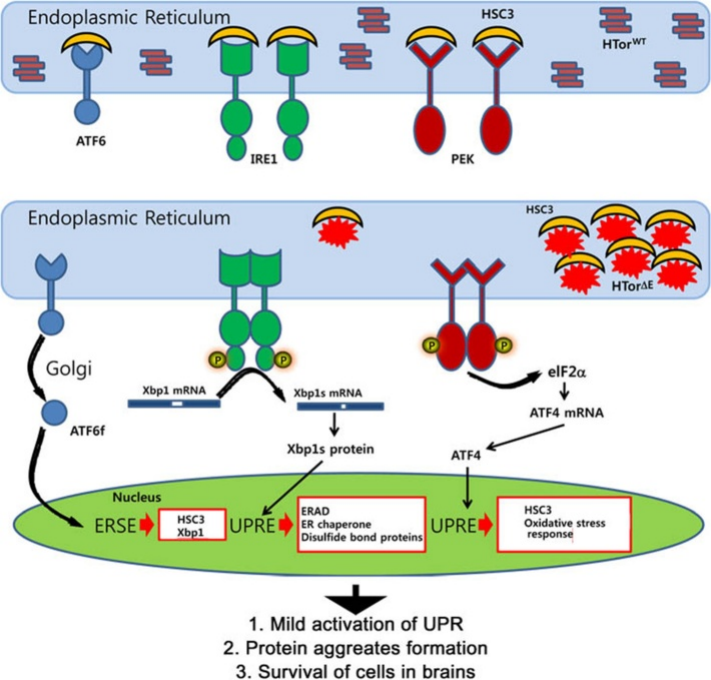
The three axes of the UPR in *Drosophila* brains activated by HTorA^ΔE^. HTorA^WT^ may not induce activation of the UPR in ER. However, HTorA^ΔE^ may induce activation of the three axes of the UPR signalling pathway by directly binding to HSC3 or by impairing protein trafficking and secretion from the ER that results in ER overload. The amounts of transcripts of the IRE1-dependent spliced Xbp1, AFT4, and ATF6 are consequently increased and initiate transcriptional up-regulation of ER-stress and UPR target genes, including HSC3, Xbp1, components of ERAD, ER chaperone, disulfide bond proteins, oxidative stress response proteins, and ATG8b in *Drosophila* brains. Consequently, the HTorA^ΔE^ flies showed an increased susceptibility to oxidative and ER stress and a prolonged UPR activation compared with the HTorA^WT^ flies.

The ER chaperone HSC3 (Figure 2 and 3) is a member of the HSP70 protein superfamily and is known to be a *Drosophila* homologue of BiP [8]. Similar to BiP and its homologues in metazoans, HSC3 has been shown to act as an ER stress regulator in flies [8]. For example, the up-regulation of HSC3 upon activation of the UPR has been shown in *Drosophila* models of ADRP [31], congenital glaucoma [47], and Parkinson disease [48]. Similar to those models, we found additional key evidence that UPR activation increased the transcript levels of the IRE1-dependent spliced form of XBP1 (Figure 3C) and the Xbp1-eGFP signal (Figure 3D). Although the amount of IRE1 and PEK mRNAs was not altered, the mRNAs of *ATF6 and ATF4* were significantly increased in the HTorA^ΔE^ brains (Figure 5A). In contrast to the other two UPR inducers that activate downstream transcription factors, Xbp1 and ATF4, ATF6 is transported to Golgi complexes where it is processed into an N-terminal 50 kDa active form that translocalises and activates the transcription of the ER stress response genes (ERSE) encoding molecular chaperones and enzymes regulating protein folding and disulfide formation (Figure 8) [5,7]. Thus, the increase of ATF6 transcripts could be interpreted as the activation of the UPR. Further confirmation of UPR activation in HTorA^ΔE^ flies was obtained by genetically reducing the expression of UPR sensors and inducers in the HTorA^ΔE^ fly brains (Figure 7). The down-regulation of *hsc3, xbp1* and *PEK* induced a significantly earlier death in the flies. Although the effects of *ATF6* down-regulation were not as pronounced as those of *hsc3, xbp1* and PEK, it also induced a significant increase in HR (Figure 7). Taken together, our results suggest that all three UPR inducers may be activated in the HTorA^ΔE^ brains.

### Increased transcripts of ERSE and UPRE in the HTorA^ΔE^ brains

One of the enigmatic observations reported from DYT1 dystonia is the absence of obvious pathological lesions in postmortem DYT1 brains [1]. Similarly, the HTorA^ΔE^ fly brains do not display any obvious neurodegenerative abnormalities [29,30], suggesting that the presence of the HTorA^ΔE^ aggregates in fly brains may not be sufficient to induce apoptosis; however, these aggregates may be sufficient to activate certain adaptive UPR signalling pathways that enable neurons to overcome the toxic effects caused by the accumulation of HTorA^ΔE^ in the NE and the ER. Interestingly, we found that the HTorA^ΔE^ flies showed some differences in sensitivity to two ER stressors, tunicamycin and DTT (Figure 4). The HTorA^ΔE^ were significantly more susceptible to tunicamycin, which inhibits the activity of UDP-N-acetylglucosamine-dolichol phosphate N-acetylglucosamine-1-phosphate transferase, but not to DTT, which inhibits the formation of disulfide bonds. This observation suggests that other unknown pathways, which were not tested in this study, may exist. In addition, among the ER chaperones, enzymes and nucleotide exchange factors that are known to respond with up-regulation upon activation of the UPR in mammalian cells [7,55], the calreticulin, P58IPK, ERp60, PDI, and dGRP170 transcripts were significantly increased (Figure 5). The increased expression of PDI at the protein level was confirmed by performing protein profiling and semi-quantification analysis in HSC3 positive microsomes (Figure 6, Additional files 4 and 5) and may explain why the HTorA^ΔE^ flies did not show DTT sensitivities as PDI activity is important for maintaining DTT resistance in yeast [56]. Thus, HTorA^ΔE^ in brains may induce a mild activation of the three branches of the UPR signalling pathway that are sufficient to induce the expression of some of the ERSE and UPR target genes (UPRE), allowing neurons to overcome and adapt to survival with HTorA^ΔE^ (Figure 8).

### Degradation of HTorA^ΔE^ by ERAD and autophagosome in *Drosophila*

Another known consequence of UPR activation is the degradation of mis-/unfolded proteins by two protein quality control pathways, including ERAD and autophagy [5,8,50]. A recent study in *Drosophila* showed that the over-expression of ERAD components suppressed the onset of retinal degeneration in the *Drosophila* ADRP model by reducing misfolded, mutated Rh-1 proteins [57]. Thus, the increased transcription levels of components of the ERAD (Figure 5C) in the HTorA^ΔE^ fly brains suggest that misfolded HTorA^ΔE^ might be metabolised by the ERAD pathway. These misfolded proteins may be exported from the ER and degraded by proteasomes in the cytosol. Interestingly, a recent study using a DYT1 neuronal cell line model demonstrated that HTorA^ΔE^ was degraded by both the proteasome and the macroautophagy-lysosome pathway and that HTorA^WT^ was mainly degraded by the macroautophagy-lysosome pathway [58]. We also provided several lines of evidence suggesting the involvement of autophagy in HTorA^ΔE^ degradation. First, we observed increased transcription levels of ATG8b, which shares 82% amino acid sequence identity and 94% similarity with ATG8a, another *Drosophila* homologue of LC3s [59]. The overexpression of ATG8a has been shown to extend the life span and resistance to oxidative stress in flies [59]. However, the consequences of ATG8b loss or gain of function mutations remain unknown. Nevertheless, both the 4.4-fold increase of ATG8b transcription levels present in the HTorA^ΔE^ brains (Figure 5E) and the increased susceptibility to autophagy inhibitors in the HTorA^ΔE^ flies (Figure 4 G-I) suggest that autophagosomes may be increased in the HTorA^ΔE^ brains and play a pivotal role in the metabolism of HTorA^ΔE^. Furthermore, in a previous study, we showed that the HTorA^ΔE^ aggregates in flies are colocalised with a lysosome marker and present within the double membrane structures at EM levels that are similar to autophago-lysosomes [29].

### HSP22, sensitivity to oxidative stress and the proteins involved in ATP synthesis, glycolysis and the TCA cycles were altered in the microsomes of HTorA^ΔE^ flies

*Drosophila* HSP22 is a small heat shock chaperone known to be involved in the regulation of cell proliferation and carcinogenesis [60]. In addition, HSP22 has been shown to be induced in response to acute heat and oxidative stress and is increased with normal aging in flies [36,46]. The over-expression of HSP22 in flies was sufficient to increase resistance to oxidative stress and extend life span [36]. Thus, transcriptional and translational increases of HSP22 in the HTorA^ΔE^-expressing brains (Figure 2 and 3) may be one of the molecular consequences for overcoming HTorA^ΔE^-induced defects that might be similar to the defects induced by acute heat and oxidative stress to brains. Considering this observation together with the activation of the UPR may help to explain why the HTorA^ΔE^-expressing flies had similar life spans to those of the HTorA^WT^-expressing flies and why they did not manifest severe dystonic symptoms under normal conditions despite the presence of protein aggregates at the NE, the ER and the mitochondria and structural defects at the synapses [29,30]. The increased HSP22 in the HTorA^ΔE^ flies might compensate for those defects caused by the presence of HTorA^ΔE^ under normal conditions. However, if the HTorA^ΔE^ flies were exposed to additional environmental stressors that usually do not induce severe defects in the HTorA^WT^ flies because of the protections afforded by HSP22 induction, the HTorA^ΔE^ flies would be susceptible to those stresses because HSP22 is no longer protecting the neurons and the brains. Indeed, we presented additional evidence to support this notion, such as the increased oxidative stress sensitivity of the HTorA^ΔE^-expressing flies (Figure 4) and the altered levels of proteins associated with ATP synthesis, glycolysis and the TCA cycle in HSC3 positive microsomes (Figure 6 G). In addition, our hypothesis and observation were further supported by recent studies showing that proteins involved in energy metabolism and the redox state were altered in human neuronal cell lines over-expressing HTorA^ΔE^ [27].

## Conclusion

In this study, we have provided several lines of evidence that strongly indicate that HtorA^ΔE^ in fly brains may induce prolonged activation of the UPR (Figure 8). Because the expression of HTorA was not induced by treatment of the PC12 cell line [61], the human kidney cell line [62], or the human glioblastoma cell line [63] with ER stressors, it may not belong to the group of ER chaperones that are up-regulated upon UPR activation. Moreover, recent studies utilising fibroblasts from DYT1 dystonia patients [17,18] suggest that HTorA is an ER molecular chaperone that regulates the trafficking and secretion of membranes and/or secretory proteins. The fact that treatment with tunicamycin was enough to induce the UPR activation but that neither HTorA^WT^ nor HTorA^ΔE^-expression in a human glioblastoma cell line did so [62,63] suggested that the expression of HTorA^ΔE^ may not be sufficient for the activation of the UPR in this tumor cell line system. How can we explain the discrepancy between our results and those reported from the DYT1 cellular models? A wide variety of tumours, such as glioblastomas, melanomas, and breast and cervical cancers activate the UPR to allow tumour cells to adapt and survive under certain conditions [4,64,65], and the expression of HTorA^ΔE^ in tumour cell lines may not be sufficient to further induce the activation of the UPR. Similar to our results, the overexpression of HTorA^ΔE^ in *C. elegans* induced the increased expression of its BiP homolog *hsp-4* tagged with GFP and IRE1-dependent spliced Xbp-1 mRNA even before tunicamycin treatment [66]. Furthermore, the decreased export of reporter proteins from DYT1 dystonia fibroblasts compared to those of normal fibroblasts [17,18] could be explained as the activation of the UPR by ER overload because the protein overload is a physiological ER stressor that could initiate UPR activation in the ER [4,49,50]. Thus, it will be intriguing to examine UPR activation and the alteration of HSP22 orthologs in the brains of DYT1 patients and available TG mouse models. Further utilisation of the HTorA^ΔE^-expressing fly brains will reveal unknown *in vivo* molecular and cellular consequences.

### Ethnic statement

We did not use any human subject or data in this study.

## Additional files

Additional file 1:
**The sequences of primers for quantitative real time reverse transcriptase PCR analysis.**
Additional file 2:
**The slope and y intercept of calibration curves and PCR efficiency calculated from slopes for qRT-PCR.**
Additional file 3:
**No differences in survival ratios between HTorAWT- and HTorA**Δ**E-expressing flies.** The p-value calculated using the log-rank test was bigger than 0.1, and the 95% confidential interval of the hazardous ratio was from 0.09092 to 2.699. Additional file 4:
**The protein identification results of LC/MSE analysis of HSC3 positive microsome fractions of the HTorA**Δ**E brains and the HTorAWT brains.**
Additional file 5:
**The label-free semi-quantification results of density gradient fraction Nos. 9–12 from the HTorA**Δ**E and the HTorAWT brains.**
Additional file 6:
**Transgenic expression of RNAi constructs for hsc3, xbp1, PEK, and ATF6 significantly reduced the amounts of their target mRNAs in fly brains.**
Additional file 7:
**Proteins identified from differentially expressed spots by a nano-HPLC ESI-quadrupole IT MS.**
Additional file 8:
**Expression of Drosophila Torsin proteins did not change expression levels of HSC3.** A) Western blot analysis results with rat anti-HSC3, mouse anti-a-Tubulin, and rabbit anti-DTor antibodies. B) Normalized amounts of HSC3 in DTor overexpressing flies were similar to the levels of control flies (Tub-Gal4/+). C) Normalized amounts of DTor in DTor overexpressing flies were 3.5-fold up-regulated compared to the levels of control flies (Tub-Gal4/+). * = *p* < 0.05. Additional file 9:
**The list of proteins with increased amounts in density gradient fraction No. 9–12 from the HTorA** ^**ΔE**^ **and the HTorA** ^**WT**^ **-expressing brains.**
Additional file 10:
**The list of proteins with decreased amounts in density gradient fraction No. 9–12 from the HTorA** ^**ΔE**^ **and the HTorA** ^**WT**^ **-expressing brains.**

## Abbreviations

2-DE: Two-dimensional electrophoresis
AAA+ ATPase: ATPases associated with a variety of cellular activities
Act-eGFP: Actin-enhanced green fluorescence protein
ADRP: Autosomal dominant retinitis pigmentosa
ATF6: Activating transcription factor 6
Cpr72: Cuticular protein 72Ec
DTT: Dithiothreitol
ΔE: A glutamic deletion
eGFP: Enhanced green fluorescence protein
ER: Endoplasmic reticulum
ERAD: ER associated degradation
ERSE: Endoplasmic reticulum stress response element
GRP78: Gluose-regulated protein 78
HR: Hazard ratio
HSC3: Heat shock cognate 3
HSP22: Heat shock protein 22
HSPA5: Heat shock 70 kDa protein 5
HTorA: Human Torsin A
IRE1: Inositol-requiring enzyme 1
KLC1: Kinesin light chain 1
LAP1: Lamina-associated polypeptide 1
LC: Liquid chromatography
LULL1: Luminal domain like LAP1
MS: Mass spectrometry
NE: Nuclear envelop
PEK: Pancreatic elF-2α kinase
PERK: Pancreatic ER kinase
QRT: Quantitative real-time
RIPA: Radio-immuno-precipitation assay
RNAi: inhibitory RNA
SCP1: Sarcoplasmic calcium binding protein 1
SDS: Sodium dodecyl sulfate
si: Small interfering
Tsf1: Transferrin 1
UPLC: Ultra pressure liquid chromatography
UPR: Unfolded protein response
UPRE: Unfolded protein response element
WT: Wild type
xbp1: x-box binding protein 1
