# Supplementary material for: The pathogenic human Torsin A in Drosophila activates the unfolded protein response and increases susceptibility to oxidative stress
Source: BMC Genomics. 2015 Apr 23;16(1):338. doi: 10.1186/s12864-015-1518-0 (PMC4415242; doi:10.1186/s12864-015-1518-0)
Supplement: Additional file 1: — The sequences of primers for quantitative real time reverse transcriptase PCR analysis. [file 12864_2015_1518_MOESM1_ESM.docx]

**Additional file 1**: The sequences of primers for quantitative real time reverse transcriptase PCR analysis.

| **Gene** | **Forward** | **Reverse** |
| --- | --- | --- |
| **RP49** | CAGTCGGATCGATATGCTAAGCTGT | TAACCGATGTTGGGCATCAGATACT |
| **HSC3** | GCTCAACCTGGATCTATTCC | TGGATGGTGACGGTGTGCTGGTTAT |
| **HSC4** | AGGTGTACGAGGGAGAGCGT | CCACTTGATGGTCTCGTTGC |
| **HSC5** | GAGCGTGAGATGGCTAACGA | AGGTCGGCAAGTCTCCTTGTT |
| **Tsf1** | CGATTGTGTGGTGGCTCTGACCAAG | AAGGACATCATCCTGAGCCCTCTGC |
| **HSP22** | GGACGTCAAGGACTACAGCGAGCTA | TATAGCCACCTTGTTCGGCCTCCTG |
| **SCP1** | AACCGTGTCGATTTTGTCGTTCGGT | ATCCAATCGAGCAGTGCTGCAATCA |
| **Xbp1+23bp** | GCACAACACCAGATGCATCAGCCAA | AGAGGGTCAGCTTTGGATGCTGCAGA |
| **Xbp1-23bp (Xbp1s)** | GCACAACACCAGATGCATCAGCCAA | TGTTGTATACCCTGCGGCAGATCCAA |
| **IRE1** | GATCCAATGGAAGCACTGGCAGCAA | TTGTGCTGAAGCTGATCTTGCCCAC |
| **PEK** | TCTGGTCATTGAACGTCATGTGCCTG | TGATTTGCTTGTCCAGGTGGGAAGC |
| **ATF6** | AACGTAATTCCACGGAAGCCCAACA | GCGACGGTAGCTTGATTTCTAGAGCC |
| **ATF4 (Cryptocephal)** | TGCGAGTCTCAGGCGTCTTCATCTT | CTGCTCGATGGTTGTAGGAGCTGG |
| **Calreticulin** | CGGCACCAAGAAGGTCCATGTGATCT | GATGAGCACCTCGTACGTGTTGTCG |
| **Calnexin 99A** | GCGTGGAAAATGGGAGGAAACCGG | GAAGTCGTCCGACTCCGTGTCCAAA |
| **ERp60** | CTTGCTGGAGTGCTCCTACTAGGCT | GTAGAACATGACCAGCGTCGTCTCG |
| **PDi** | TCTGCTTCCAAGATCTTCGGTGGCT | ACAGGATGTCGTCGCGGTACTTCTT |
| **P58IPK** | CCTTGCCATTGAGTGACCTGCTCAT | TGCCCAGCTCCAGATGGTTCTCAAT |
| **GP93 (dGRP94)** | TACCTGAGCTTCATTCGTGGCGTCG | GCGGACCAGCTTCTTCTTGATCACC |
| **CG2918 (dGRP170)** | CAGTCTGGACGTGATCAACCAGGTG | ACTGCACCCATAGTGGCGGATTCAT |
| **CG10420 (dSIL1)** | ACCTCGGGTACACAGGCTCTGATC | AGAGTCACTACCTTCGCCTTGCTCC |
| **Sip3 (Hrd1)** | GCACGGCCATCAAGTATGTGCTTCA | GCTCTGTATATAGCAGGAACACGGCCT |
| **Hrd3** | GCTGTGAGAAGGCGCTGATCCACTA | CCAGCAGTCTTACCCGATGCACAAC |
| **Herp** | CTTACGCGCAGTACATGCAGCAGTT | CAGCTGCTCCTGCCACTTGTTGTAC |
| **Derlin-1** | CGAAGTCCGGCTGATTACCTGTACC | TGGCACCAGATATACGTGATGGCCA |
| **EDEM1** | CAATCCGGCACAAGCACTACCATGG | CCTCGCGTATCTGCTCGAAGTTGCT |
| **EDEM2** | CATGCACAGGCCACATGCTAAGTCC | TGAAGGTGTGCATCTGCATGACCTGA |
| **Atg1** | GGATCATTGGGCTCGATTGGTTCGG | GGCTCCTGTGTCCAGCAGACTATG |
| **Atg2** | TTCACGCATGGACAAGTTCGGTGTC | GATGGAGGCCTAAGCAGGTCCACTT |
| **Atg3 (Aut1)** | CAAGCCACTGACCGTGGAACAGATG | CACAGAGGCCATATTGGGACCTGGA |
| **Atg4** | CGACTTACATTTGGGACGCGACTGG | ACTCTCCCATCTGGGCAATCTGGTG |
| **Atg5** | TGGTGCCTCACCATTCACTTCTCCA | TGCCTCCTTCAGGCAGGACATGTAG |
| **Atg6** | CAGATCGCCTACTCGAAACAGCAGC | AGTCCACGGATACAGAGGGCAATCG |
| **Atg7** | CGACATGGCACTACGCGTAAGGAAG | CGGTCTTTGAGCGAATTTCCAGGGG |
| **Atg8a** | CTCAGCAACGCATCGATATTCGGCA | CGAAGGCGTGCTCCTCCTTGTATTG |
| **Atg8b** | CCGATGAGAACGTCTATGGACGGCA | CTGGTTAGCTGGCTAGGAGCAGTCA |
| **Atg9** | ACTGCTCTACGGACTCTGTGGTGTG | GTGCAAATAGAGGGCACTGAGGCAC |
| **Atg12** | ACAGTCGGCTGGATACAGACGTTCA | TCCCATTGGTTCCATGGCACTCGTA |
| **Atg13** | GTGGTGGGCTCCAAATGAGCAACTG | GCCTCTCTCCATATTGACCGCCCTT |
| **Atg18** | TTGATTGCCTCGCAGGATGGGTACT | GCCGCAGGTCGTGCCTCTTTATTAG |
