# Supplementary material for: The pathogenic human Torsin A in Drosophila activates the unfolded protein response and increases susceptibility to oxidative stress
Source: BMC Genomics. 2015 Apr 23;16(1):338. doi: 10.1186/s12864-015-1518-0 (PMC4415242; doi:10.1186/s12864-015-1518-0)
Supplement: Additional file 2: — The slope and y intercept of calibration curves and PCR efficiency calculated from slopes for qRT-PCR. [file 12864_2015_1518_MOESM2_ESM.docx]

**Additional file 2:** The slope and y intercept of calibration curves and PCR efficiency calculated from slopes for qRT-PCR.

| **Gene** | **Slope, y intercept, and γ^2^ of calibration curves** | **PCR efficiency** |
| --- | --- | --- |
| **RP49** | y = -3.454x + 17.06, γ^2^ = 0.999 | 0.94758 |
| **HSC3** | y = -3.528x + 19.05, γ^2^ = 0.999 | 0.92064 |
| **HSC4** | y = -3.468x + 14.43, γ^2^ = 0.999 | 0.94219 |
| **HSC5** | y = -3.114x + 17.80, γ^2^ = 0.999 | 1.09439 |
| **Tsf 1** | y = -3.134x + 14.14, γ^2^ = 0.999 | 1.08454 |
| **HSP22** | y = -3.546x + 18.67, γ^2^ = 0.998 | 0.91405 |
| **SCP1** | y = -3.453x + 24.10, γ^2^ = 1.000 | 0.94795 |
| **Xbp1+23bp** | y = -3.654x + 17.88, γ^2^ = 0.999 | 1.03167 |
| **Xbp1-23bp (Xbp1s)** | y = -3.422x + 21.31, γ^2^ = 0.998 | 0.91405 |
| **IRE1** | y = -3.373x + 21.06, γ^2^ = 0.998 | 0.97884 |
| **PEK** | y = -3.474x + 23.97, γ^2^ = 0.996 | 0.94023 |
| **ATF6** | y = -3.131x + 22.67, γ² = 0.996 | 1.08601 |
| **ATF4 (Cryptocephal)** | y = -3.585x + 18.21, γ^2^ = 0.999 | 0.90082 |
| **Calreticulin** | y = -3.512x + 17.63, γ^2^ = 0.999 | 0.92636 |
| **Calnexin 99A** | y = -3.583x + 19.83, γ^2^ = 0.998 | 0.90126 |
| **ERp60** | y = -3.535x + 19.49, γ^2^ = 0.995 | 0.91816 |
| **PDi** | y = -3.275x + 18.15, γ² = 0.999 | 1.01996 |
| **P58IPK** | y = -3.388x + 23.29, γ² = 0.999 | 0.97303 |
| **GP93 (dGRP94)** | y = -3.510x + 20.31, γ^2^ = 0.999 | 0.92708 |
| **CG2918 (dGRP170)** | y = -3.518x + 21.12, γ^2^ = 0.999 | 0.92410 |
| **CG10420 (dSIL1)** | y = -3.373x + 21.06, γ^2^ = 0.998 | 0.97884 |
| **Hrd1 (Sip3)** | y = -3.236x + 21.86, γ^2^ = 0.995 | 1.03684 |
| **Hrd3** | y = -3.257x + 21.23, γ^2^ = 0.997 | 1.02752 |
| **Herp** | y = -3.500x + 21.53, γ^2^ = 0.999 | 0.93070 |
| **Derlin-1** | y = -3.342x + 20.83, γ^2^ = 0.987 | 0.99169 |
| **EDEM1** | y = -3.255x + 21.61, γ^2^ = 0.997 | 1.02871 |
| **EDEM2** | y = -3.175x + 24.18, γ^2^ = 0.994 | 1.06482 |
| **Atg1** | y = -3.563x + 21.24, γ² = 0.999 | 0.90837 |
| **Atg2** | y = -3.219x + 22.89, γ² = 0.996 | 1.04450 |
| **Atg3 (Aut1)** | y = -3.524x + 21.39, γ² = 0.999 | 0.92182 |
| **Atg4** | y = -3.269x + 21.84, γ² = 1.000 | 1.02257 |
| **Atg5** | y = -3.480x + 23.80, γ² = 0.998 | 0.93790 |
| **Atg6** | y = -3.386x + 23.34, γ² = 0.998 | 0.97366 |
| **Atg7** | y = -3.253x + 22.85, γ² = 0.999 | 1.02946 |
| **Atg8a** | y = -3.503x + 17.19, γ² = 0.999 | 0.92936 |
| **Atg8b** | y = -3.270x + 30.00, γ² = 0.997 | 1.02214 |
| **Atg9** | y = -3.339x + 22.67, γ² = 0.999 | 0.99264 |
| **Atg12** | y = -3.390x + 22.91, γ² = 0.998 | 0.97236 |
| **Atg13** | y = -3.424x + 22.75, γ² = 0.997 | 0.95898 |
| **Atg18** | y = -3.398x + 22.84, γ² = 0.999 | 0.96909 |
