# Supplementary material for: The pathogenic human Torsin A in Drosophila activates the unfolded protein response and increases susceptibility to oxidative stress
Source: BMC Genomics. 2015 Apr 23;16(1):338. doi: 10.1186/s12864-015-1518-0 (PMC4415242; doi:10.1186/s12864-015-1518-0)

Additional file 3: No differences in survival ratios between HTorA^WT^- and HTorA^ΔE^-expressing flies. The p-value calculated using the log-rank test was bigger than 0.1, and the 95% confidential interval of the hazardous ratio was from 0.09092 to 2.699


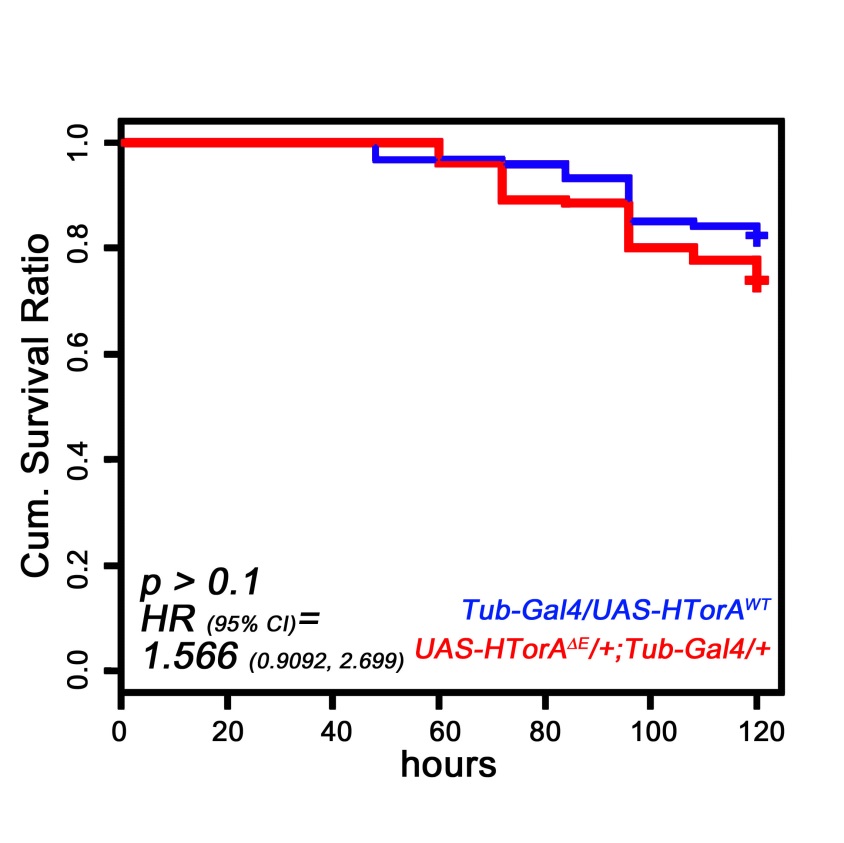

Supplement: Additional file 3: — No differences in survival ratios between HTorAWT- and HTorAΔE-expressing flies. The p-value calculated using the log-rank test was bigger than 0.1, and the 95% confidential interval of the hazardous ratio was from 0.09092 to 2.699. [file 12864_2015_1518_MOESM3_ESM.docx]
