# Supplementary material for: The pathogenic human Torsin A in Drosophila activates the unfolded protein response and increases susceptibility to oxidative stress
Source: BMC Genomics. 2015 Apr 23;16(1):338. doi: 10.1186/s12864-015-1518-0 (PMC4415242; doi:10.1186/s12864-015-1518-0)

Additional file 6: Transgenic expression of RNAi constructs for hsc3, xbp1, PEK, and ATF6 significantly reduced the amounts of their target mRNAs in fly brains. (a) When hsc3-RNAi constructs were expressed in Drosophila brains, only 27.17% hsc mRNA was present compared with control brains. (b) Transgenic expression of xbp1-RNAi constructs in brains were significantly reduced unspliced (xbp1+23bp) and spliced (xbp1-23bp) xbp1 mRNA up to 71.2% and 54.3%, respectively, compared to those of wild type controls. (c) Expression of PEK-RNAi in brains reduced expression of PEK and ATF4 to 29.0% and 76.7%, respectively, compared to those of wild type controls. (d) Only 47.0% of ATF6 mRNA was present in brains expressing ATF6-RNAi compared to those of control brains. ***, p < 0.005.


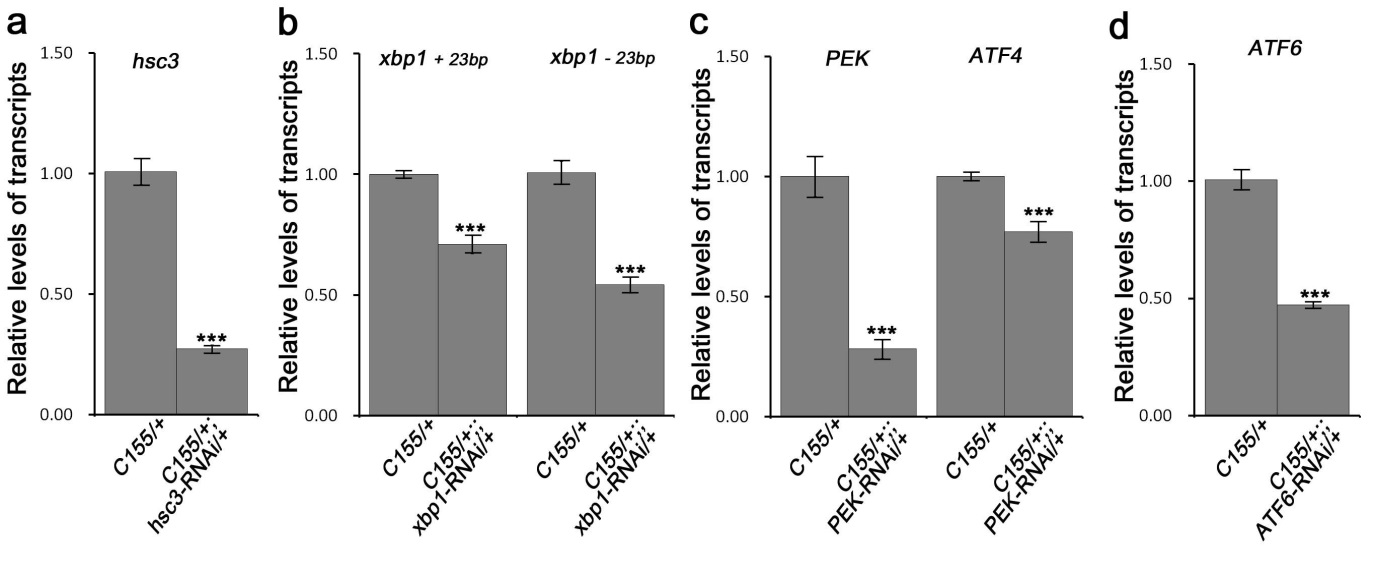

Supplement: Additional file 6: — Transgenic expression of RNAi constructs for hsc3, xbp1, PEK, and ATF6 significantly reduced the amounts of their target mRNAs in fly brains. [file 12864_2015_1518_MOESM6_ESM.docx]
