# Supplementary figures and images for: The pathogenic human Torsin A in Drosophila activates the unfolded protein response and increases susceptibility to oxidative stress
Source: BMC Genomics. 2015 Apr 23;16(1):338. doi: 10.1186/s12864-015-1518-0 (PMC4415242; doi:10.1186/s12864-015-1518-0)

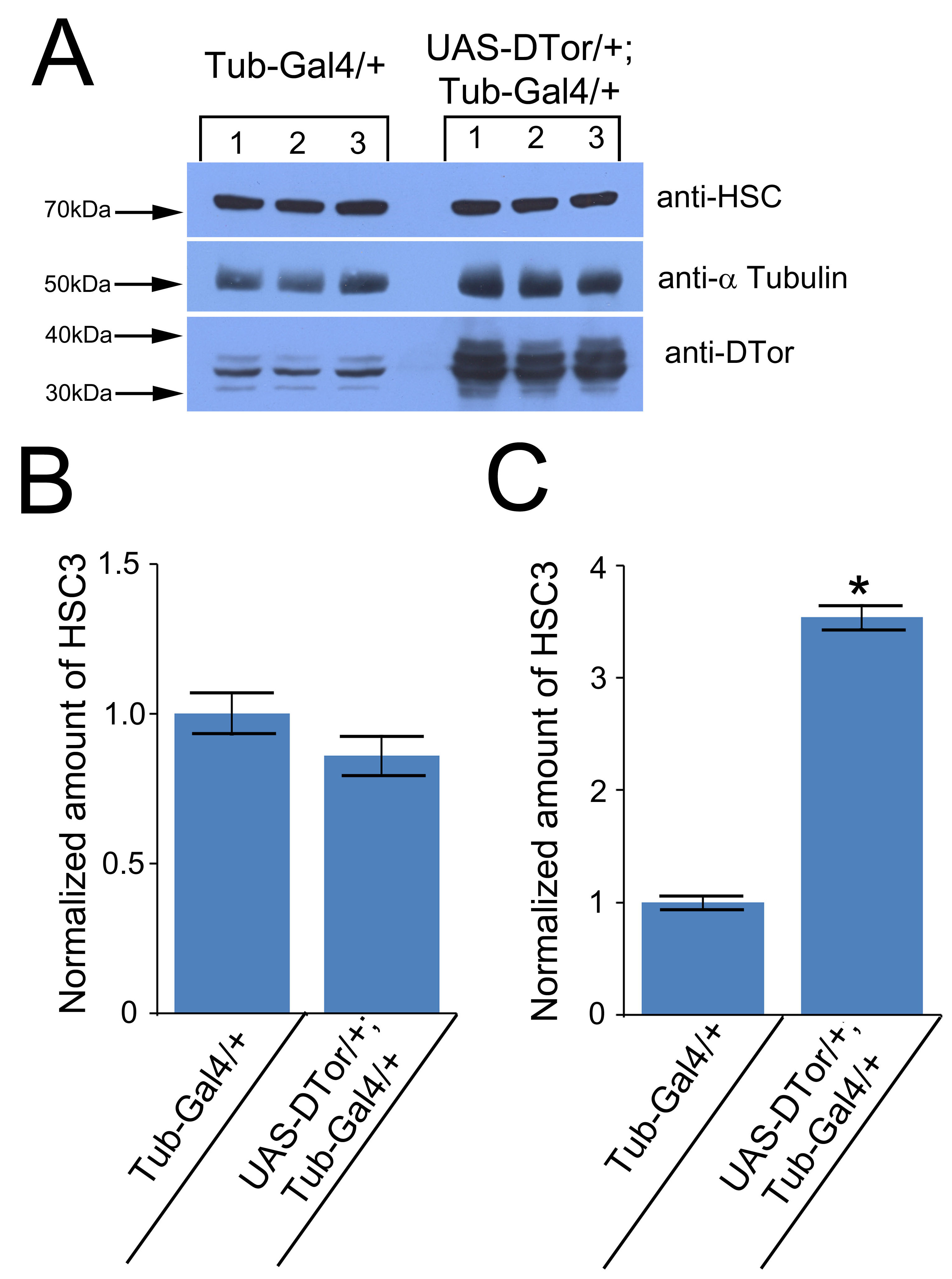

Supplement: Additional file 8: — Expression of Drosophila Torsin proteins did not change expression levels of HSC3. A) Western blot analysis results with rat anti-HSC3, mouse anti-a-Tubulin, and rabbit anti-DTor antibodies. B) Normalized amounts of HSC3 in DTor overexpressing flies were similar to the levels of control flies (Tub-Gal4/+). C) Normalized amounts of DTor in DTor overexpressing flies were 3.5-fold up-regulated compared to the levels of control flies (Tub-Gal4/+). * = p < 0.05. [file 12864_2015_1518_MOESM8_ESM.jpeg]
