# Supplementary material for: The pathogenic human Torsin A in Drosophila activates the unfolded protein response and increases susceptibility to oxidative stress
Source: BMC Genomics. 2015 Apr 23;16(1):338. doi: 10.1186/s12864-015-1518-0 (PMC4415242; doi:10.1186/s12864-015-1518-0)
Supplement: Additional file 9: — The list of proteins with increased amounts in density gradient fraction No. 9–12 from the HTorA ΔE and the HTorA WT -expressing brains. [file 12864_2015_1518_MOESM9_ESM.docx]

Additional file 9: The list of proteins with increased amounts in density gradient fraction No. 9-12 from the HTorA^ΔE^ and the HTorA^WT^-expressing brains.

|  | Lane 9 | | Lane 10 | | Lane 11 | | Lane 12 | |
| --- | --- | --- | --- | --- | --- | --- | --- | --- |
|  | Accession No. | Gene name | Accession No. | Gene name | Accession No. | Gene name | Accession No. | Gene name |
| Proteins increase (HTorA^WT^/  HTorA^ΔE^ < 0.5) or detected  in  HTorA^ΔE^ microsome | O62526  Q26365  Q7KML1  Q9VER8  P05303  Q9VA91  P50887  Q9W229 | Ant2  sesB  GH07626  CG14906  Ef1α100E  RpS7  RpL22  RpS24 | P02844  Q8T4T4  A5XCL5  O62526 | Yp2  CG5389  UGP  Ant2 | P05303  Q7KN97  B6IDT3  C7LAA3  P10676  Q9VAC1  P53501  Q9W401  Q7K569  Q8T4C4  P006603  P11147  P02844  Q9VM14  Q8MR70  P29844  Q94523  Q05825  Q7KML1  O62619  Q94920  Q6!WP8  Q29QY4  P08736  Q9VVC5  Q27331  Q3ZAJ5  P19889  Q917S8  A8JRB8  P22808  P12024  Q7K5K3  Q0XTL9  Q9Y119  Q94511  Q9VER8  Q9VA91  B7Z061  P07486  P07487  Q9VFF0 | Ef1α100E  CG1516  CG3523  Act5C  ninaC  CG7920  Act57B  kdn  Gpo1  CG5389  αTub84B  Hsc4  Yp2  CG5261  K05816  HSC3  SdhA  ATPsyn β  GH07626  PyK  porin  RE70805p  IP15846p  Ef1α48D  Nc73EF  Vha68  Arp53D  RpLP0  Ade5  CG5028  vnd  chp  CG11876  GlyP  Tps1  ND75  CG14906  RpS7  Pdh  Gapdh1  Gapdh2  CG3731 | Q9W5W8  P12024  Q24439  Q05825  P02574  P35301  Q9VGQ1  Q7KN97  P06603  P10987  C7LAA3  P13607  P29844  P11147  Q7JR58  Q9VL7O  P31409  Q27331  Q29QE3  P19889  Q7KMP8  P05303  Q8MR70  P08841  Q9VNW6  P10676  Q9VHJ8  P54399  P36179  Q7KML1  P54385  Q9W1H8  Q6NKL9  P50887  Q8IPE8  P10981  Q9XTL9  P08736  Q9W514  Q9VSI6  Q9VWH4  Q8IGI6  P29310  Q94920  O01666  Q9VRL0  Q94516  C6TP50  Q8IQQ0  Q8MST5  Q9VA91  B7Z061  Q26365  P41042  P54611  B6IDT3  Q9VFF0  Q7KN62  Q9VSW1 | CG9577  chp  Oscp  ATPsyn β  Act79B  blw  Act57B  CG5214  αTub84B  kdn  Rfabg  ATPase α  Hsc3  Hsc4  CG6543  Yip2  Vha55  Vha68  Yp1  RpLP0  Rpn9  Ef1α100E  k05816  βTub60D  CG7470  ninaC  skap  Pdi  PP2A  GH07626  Gdh  Thiolase  RH57795  RpL22  CG4389  Act87E  GlyP  Ef1α48D  Rpt4  Idh  CG12233  RH09189  14-3-3 zeta  porin  ATPsyn γ  CG4769  ATPsyn β  kdn  Nc73EF  βTub97EF  RpS7  Pdh  sesB  RpS4  Vha26  CG3523  CG3731  TER94  UGP |
| Proteins decreased (HTorA^WT^/HTorA^ΔE^ >2.0) or not- detected from  HTorA^ΔE^  microsomes | Q24439  P06607  P83967  P02844  P02843  Q8T4C4  Q9VT32  P07764  Q94920 | Oscp  Yp3  Act88F  Yp2  Yp1  CG5389  CG6767  Ald  Porin | P2043  Q24251  Q9VVC5  Q9VWH4  P06603  O62619  Q24388  Q9VEB1  B5RJK7 | Yp1  ATPsyn d  Nc73EF  CG12233  αTUB84B  PyK  Lsp2  CG7998  Yp2 | C8VUY7  Q9VT32  Q9V397  D3DMQ1  P41042  P02574 | ATPase α  CG6767  CH12558  Yp3  RpS4  Act79B | P02844  Q24388  Q4V3F7  Q8T3P0  Q9VHN7  P91938  Q9VW68  Q9W457  P20228  Q9VCK6  Q9VC18  P20228  Q9VIT9  P02572 | Yp2  Lsp2  GH10614  CG7145  CG8036  CG7461  Trxr1  CG7433  CG3011  Gad1  CG10184  CG11089  TepIV  Act42A |
