# Supplementary material for: The pathogenic human Torsin A in Drosophila activates the unfolded protein response and increases susceptibility to oxidative stress
Source: BMC Genomics. 2015 Apr 23;16(1):338. doi: 10.1186/s12864-015-1518-0 (PMC4415242; doi:10.1186/s12864-015-1518-0)
Supplement: Additional file 10: — The list of proteins with decreased amounts in density gradient fraction No. 9–12 from the HTorA ΔE and the HTorA WT -expressing brains. [file 12864_2015_1518_MOESM10_ESM.docx]

Additional file 10: The list of proteins with decreased amounts in density gradient fraction No. 9-12 from the HTorA^ΔE^ and the HTorA^WT^-expressing brains.

|  | Lane 9 | | Lane 10 | | Lane 11 | | Lane 12 | |
| --- | --- | --- | --- | --- | --- | --- | --- | --- |
|  | Accession No. | Gene name | Accession No. | Gene name | Accession No. | Gene name | Accession No. | Gene name |
| Proteins decreased (HTorA^WT^/HTorA^ΔE^ >2.0) or not- detected from  HTorA^ΔE^  microsome | Q24439  P06607  P83967  P02844  P02843  Q8T4C4  Q9VT32  P07764  Q94920 | Oscp  Yp3  Act88F  Yp2  Yp1  CG5389  CG6767  Ald  Porin | P2043  Q24251  Q9VVC5  Q9VWH4  P06603  O62619  Q24388  Q9VEB1  B5RJK7 | Yp1  ATPsyn d  Nc73EF  CG12233  αTUB84B  PyK  Lsp2  CG7998  Yp2 | C8VUY7  Q9VT32  Q9V397  D3DMQ1  P41042  P02574 | ATPase α  CG6767  CH12558  Yp3  RpS4  Act79B | P02844  Q24388  Q4V3F7  Q8T3P0  Q9VHN7  P91938  Q9VW68  Q9W457  P20228  Q9VCK6  Q9VC18  P20228  Q9VIT9  P02572 | Yp2  Lsp2  GH10614  CG7145  CG8036  CG7461  Trxr1  CG7433  CG3011  Gad1  CG10184  CG11089  TepIV  Act42A |
